# Zn(II) Heteroleptic Halide Complexes with 2-Halopyridines: Features of Halogen Bonding in Solid State

**DOI:** 10.3390/molecules26113393

**Published:** 2021-06-03

**Authors:** Mikhail A. Vershinin, Marianna I. Rakhmanova, Alexander S. Novikov, Maxim N. Sokolov, Sergey A. Adonin

**Affiliations:** 1 Nikolaev Institute of Inorganic Chemistry SB RAS, Lavrentieva St. 3, 630090 Novosibirsk, Russia; mvershinin@ngs.ru (M.A.V.); rakhmanova_m@mail.ru (M.I.R.); caesar@niic.nsc.ru (M.N.S.); 2 Institute of Chemistry, Saint Petersburg State University, Universitetskaya Nab. 7–9, 199034 Saint Petersburg, Russia; ja2-88@mail.ru

**Keywords:** zinc, halide complexes, halogen bonding, non-covalent interactions

## Abstract

Reactions between Zn(II) dihalides and 2-halogen-substituted pyridines 2-XPy result in a series of heteroleptic molecular complexes [(2-XPy)_2_ZnY_2_] (Y = Cl, X = Cl (**1**), Br (**2**), I (**3**); Y = Br, X = Cl (**4**), Br (**5**), I (**6**), Y = I, X = Cl (**7**), Br (**8**), and I (**9**)). Moreover, **1**–**7** are isostructural (triclinic), while **8** and **9** are monoclinic. In all cases, halogen bonding plays an important role in formation of crystal packing. Moreover, **1**–**9** demonstrate luminescence in asolid state; for the best emitting complexes, quantum yield (QY) exceeds 21%.

## 1. Introduction

Halogen bonding (XB) is a specific kind of non-covalent interaction (according to IUPAC, it “occurs when there is evidence of a net attractive interaction between an electrophilic region associated with a halogen atom in a molecular entity and a nucleophilic region in another, or the same, molecular entity” [1]), which was intensively investigated in recent years. Attention on this phenomenon is driven by both fundamental interest and its consideration as an additional tool for directed design of functional supramolecular systems. Indeed, formation of XB can influence different properties of compounds—in solution and, especially, in solid state. These include, in particular, luminescence, which can be amplified [2,3,4,5,6] by XB, solvatochromism [7,8], catalytic activity [9,10,11], and even odor [12]. As a result, “XB strategy” can be widely applied in materials science (especially in development of various sensors [13,14,15]).

Among the great variety of building blocks able to form XB, there is a specific group of neutral complexes with a general formula [M^II^(XPy)_2_Y_2_], where XPy is halogen-mono- or poly-substituted pyridine and Y is halide ligand. In most of cases [16,17,18,19,20,21,22,23,24,25], there appears: XB between X and Y in a solid state [26,27,28,29,30,31], featuring diverse connectivity patterns [32,33,34]; the prominence of these interactions for this class even inspired its “visualization” by experimental charge density determination [33], which is not common in XB studies. According to the Cambridge Structural Database (CSD), such complexes are best represented for M = Cu [22,24,26,35]. Interestingly, there are noticeably less examples for M = Zn(II), and there are at least two surprising facts. First, earlier works concentrated on structural aspects [36] of [Zn(XPy)_2_Y_2_], but not on their luminescent properties, while the latter were studied for relevant complexes with non-halogen-substituted pyridines (for example, 3- and 4-picolines [37]), demonstrating moderate quantum yields (up to 16% [37]). Second, complexes with structurally simple, commercially available X-Py—2-chloro, 2-bromo- or 2-iodopyridine—remained overlooked (or, at least, their structures were not presented in papers; only very recently, few relevant structures were deposited to CSD by C. Hu (no. 1984259–1984263)).

The strategy of our work focused on two points. First, we prepared the whole [(2-XPy)_2_ZnY_2_] series in order to see how the differences in X or Y affect the features of crystal packing and XB patterns in solid state, additionally examining the XB by theoretical methods. Second, we investigated the luminescent behavior of these complexes in comparison with other [ZnL_2_Y_2_] compounds, in particular, with [(2-MePy)_2_ZnX_2_] (X = Cl (**10**), Br (**11**), and I (**12**)). Both tasks were fulfilled; results are presented below.

## 2. Results and Discussion

According to XRD data, **1**–**7** are isostructural to each other (triclinic, see the experimental section), but not to **8** and **9**. This fact was not obvious at the initial stage of our work, considering overall situation for [Py_2_M^II^X_2_] complexes. For example, all four compounds in the [(3-XPy)_2_CuY_2_] series (X = Cl, Br; Y = Cl, Br) [25,26,38] are monoclinic and isostructural to each other while the corresponding [(3-IPy)_2_CuX_2_] (X = Cl, Br) [27,29] crystallize in the least symmetric group (triclinic); a similar feature can be noted for [(3-XPy)_2_PdCl_2_] (those are isostructural for X = Cl and Br, but not I) [18]. From this point of view, the family of **1**–**7** is especially interesting since it allows the direct comparison of XB energies for different pairs of halogens (see below).

**Figure 1:**
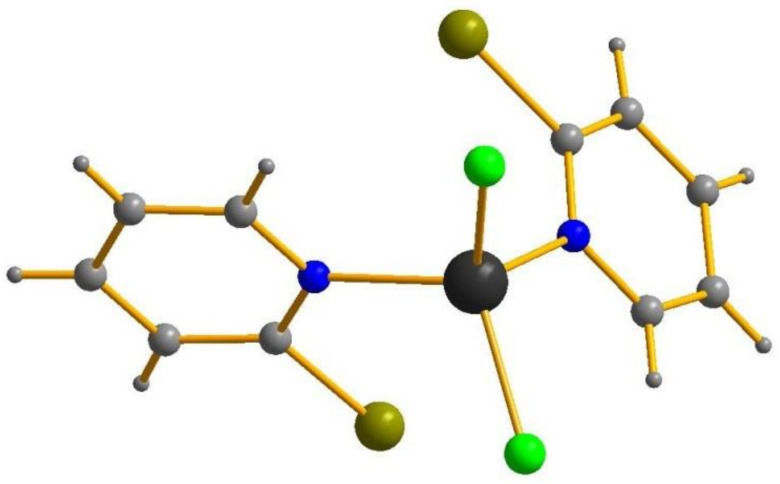
Structure of **2**. Here and below Zn black, Cl light green, Br olive–green, N blue, C grey.

In all cases, Zn(II) retains the tetrahedral coordination environment (Figure 1). Zn-Y and Zn-N bond lengths are given in Table 1; it can be seen that their differences are negligible.

**Figure 2:**
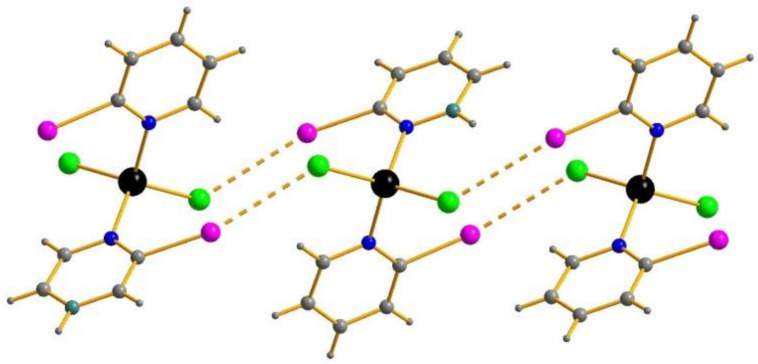
I···Cl contacts (dashed) in the structure of **3**. I purple.

A noteworthy observation can be made while analyzing the X···Y distances (d_XY_) in **1**–**7**. The identity of crystal packing results in the identity of hypothetical X···Y interaction patterns: suggesting their existence, one-dimensional chains must form via the contacts of halide ligands and halogen atoms of 2-XPy units (Figure 2) in all cases. However, comparison of d_XY_ with the sums of the corresponding Bondi’s van der Waals radii (S_XY_) [39,40] (Table 1) indicate that the situation is actually rather different. In the complexes with 2-chloropyridine, d_XY_ slightly (by < 0.1 Å) exceeds S_XY_ with only two exceptions: in **4** and **7**, the S_XY_-d_XY_ values are +0.018 and −0.027 Å, respectively. The highest (S_XY_-d_XY_) were observed for the complexes with 2-IPy (0.316 and 0.280 for **3** and **6**, respectively). On the one hand, these facts confirm that 2-IPy is a better XB donor than 2-BrPy, and especially 2-ClPy (as it was noted in related Co(II) complexes [41]). On the other hand, this allows us to draw the hypothesis that X···Y interactions can also be present in the structures where S_XY_ < d_XY_ (such situations were described earlier [42,43]); to verify this, we performed DFT calculations (see below).

**Figure 3:**
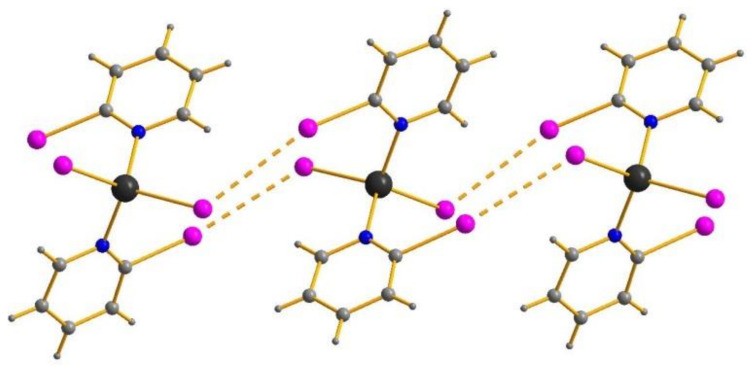
I···I contacts (dashed) in the structure of **9**.

Moreover, **8** and **9** represent the isostructural pair. Surprisingly, preparation of their single crystals of sufficient quality became a non-trivial task: after numerous XRD experiments, we succeeded in isolation of **9** to give *R_int_* = 0.037 (see Table S1 in Supplementary Materials), though revealing strong residual density peaks. For **8**, the best result was 0.080; this experiment confirmed that: 1) the crystal contains only [(2-BrPy)_2_ZnI_2_] units and 2) its cell parameters (8.7876, 14.8613, 11.7322 with β = 93.308°) are very similar to those in **9** (Table S1, Supplementary Materials), allowing judging on phase purity of **8**. However, the quality of SCXRD data does not allow us to consider that interatomic distances can be estimated reliably in this case (this explains the “missing line” in Table 1). Interestingly, despite significant differences in symmetry and cell parameters, the patterns of X···Y interactions (Figure 3) in **9** and, very likely, **8**, are very similar to those in **1**–**7** (it can be seen comparing C-X-Y and Zn-Y-X angles, Table 1). The interatomic distances found in the structures reported by C. Hu (no. 1984259–1984263 in CSD, see above; all determined at room temperature) match well with those found in corresponding compounds of the **1**–**9** series.

Comparison of crystallographic data for **1–9** and other structurally related compounds (neutral [(2-XL)_2_MY_2_] with tetrahedral M, L = *o-*halogen-substituted 6-membered unit and Y = halide) allows detecting that all those are isostructural to either **1**–**7** or, in one case, to **8** and **9**. Interestingly, the same situation was observed for o-methyl-substituted derivatives (Table 2) with only two exceptions: [(2-MePy)_2_MI_2_] (M = Zn(II) and Cd(II)) are isostructural to each other, but not to **8** and **9**.

**Figure 4:**
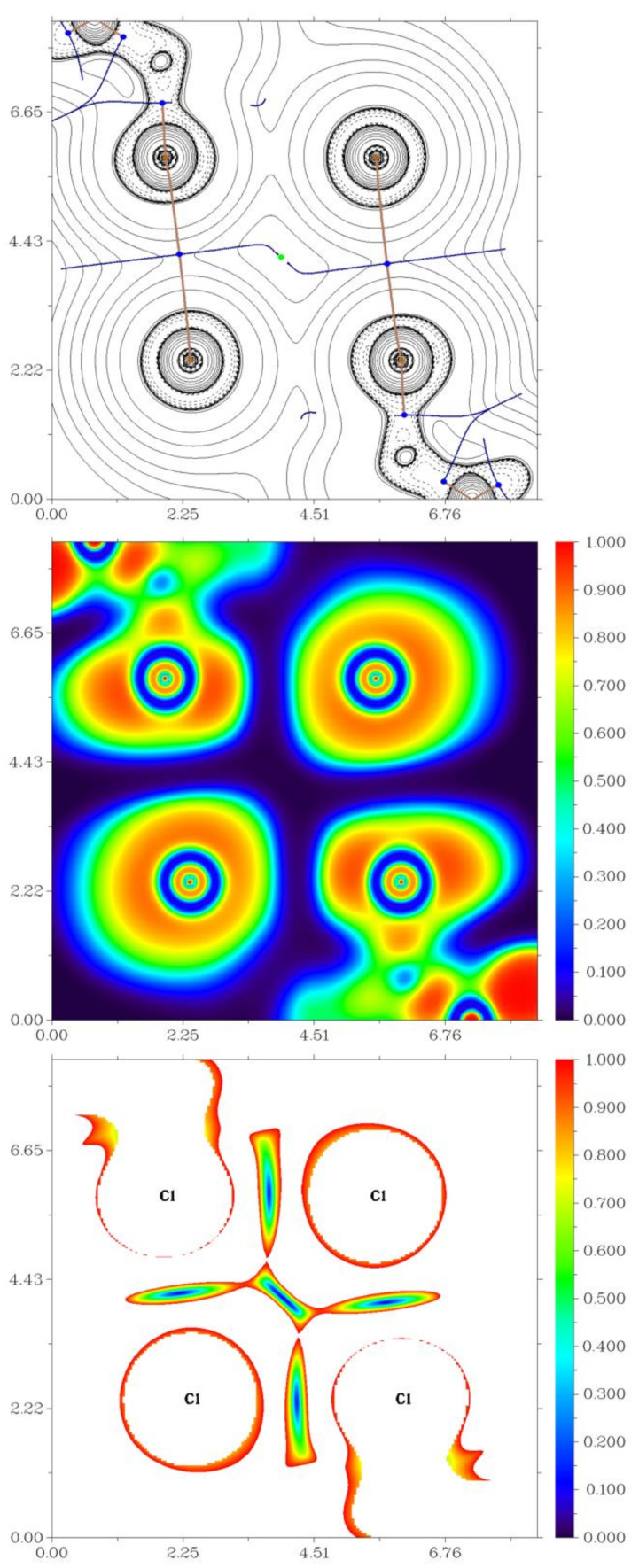
Contour line diagram of the Laplacian of electron density distribution ∇^2^ρ(**r**), bond paths, and selected zero-flux surfaces (top panel), visualization of electron localization function (ELF, center panel), and reduced density gradient (RDG, bottom panel) analyses for intermolecular non-covalent interactions Cl···Cl in **1**. Bond critical points (3, −1) are shown in blue, nuclear critical points (3, −3)—in pale brown, cage critical points (3, +3)—in light green, bond paths are shown as pale brown lines, length units—Å, and the color scale for the ELF and RDG maps are presented in a.u.

For estimation of the energies of hypothetic XB in **1**–**9**, we applied an approach that was successfully used by us [48,49,50,51] and other researchers [52,53,54,55,56,57] for relevant supramolecular systems: atomic coordinates for model clusters were obtained by XRD and used for DFT calculations and computation of the properties of electron density in the bond critical points (3, −1) within the Quantum Theory of Atoms in Molecules (QTAIM) method, “as is”, without optimization (we did not use fully relaxed geometries because we were interested in evaluating the interactions as they stood in the solid state instead of finding the most global minimum energy of the complex, see Computational Details section for details). Results are summarized in Table 3; their graphical visualizations are presented in Figure 4 and Figure S23, Supplementary Materials. As follows from these data, bond critical points (3, −1) can be found even in the case of **7** where the halogen···halogen distance exceeds the sum of van der Waals radii by over 0.1 Å. This observation provides an additional argument in favor of the point of view that the “straightforward” approach towards description of non-covalent interactions in the crystalline state, based exclusively on van der Waals radii, may be misleading in certain cases. Even though such “non-conventional” contacts seem rather weak (≈1 kcal/mol), their presence can affect the packing. Moreover, results of calculations confirm the conclusions made by us earlier: [41]; the ability of coordinated 2-XPy to serve as XB donors increase in Cl < Br < I row, so that the highest energies were found for I···Cl interactions in **3** (3.8 kcal/mol).

**Figure 5:**
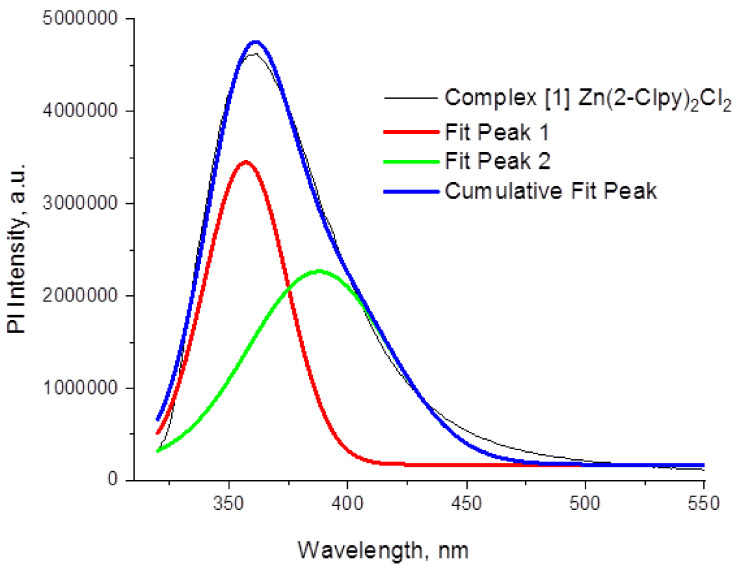
Emission spectra of **1** and its decomposition on two and three Gauss components, T = 298 K, λex = 350 nm. Gauss fit was performed in eV scale and then spectra were reported in nm scale. Conversion between eV and nm scales was performed with account of λ_2_ factor [61].

As follows from PXRD and element analysis data (see Supplementary Materials), **1**–**9** can be prepared as pure phases, making investigation of their luminescent properties possible. All complexes reveal emission in the blue–green range when irradiated with UV (spectra for **1** are presented on Figure 5, for all other complexes—in Supplementary Materials). A short summary is given in Table 4.

**1**–**12** display excitation peaks in the range of 280–320 nm (see Supplementary Materials), and one distinct weaker band at 350–420 nm. The emission spectra of **4** show three peaks and the other nine complexes give only two. No emission could be detected for the observed **8** and **12**. The Gaussian decomposition of the PL spectra (see Supplementary Materials) allows suggesting a superposition of blue and green peaks.

The most intensive peaks are between 360 and 520 nm. Emission observed in **7** and **9** is found to be red-shifted with respect to those of bromo- and chloro complexes, following the trend I > Br > Cl. This effect in emission maxima is associated to an increasing electron-donating nature (I^−^ < Br^−^ < Cl^−^) of the halide ligands. For iodide complexes, emission is fundamentally different from those for Cl- and Br-containing species.

All complexes reveal fluorescence behavior with lifetime decay of few nanoseconds, except the complexes for which it was not possible to measure lifetime decay due to very poor emissive response when excited with the pulsed source. The compounds show a mono exponential fit of each components of the decay curve, with lifetimes in the range 0.2–8.2 ns. The values reported are the average of three independent determinations for each sample. A decrease of emission quantum yield from chloride to iodide in the series of complexes is likely a consequence of the increase in the atomic number of the halogen atom bound to the zinc center; this causes a spin–orbit enhancement, which in turn favors intersystem crossing.

## 3. Materials and Methods

All reagents were obtained from commercial sources and used as purchased. Solvents were purified according to the standard procedures. Complexes **10**–**12** were prepared similarly to the procedure described earlier [46], (corresponding Zn(II) halide and 2-MePy (1:2) in ethanol) and identified by PXRD and element analysis data. All experiments were performed at room temperature.

### 3.1. Synthesis of ***1***–***9***

In all cases, 0.25 mg of ZnCl_2_·4H_2_O (53 mg), ZnBr_2_ (56 mg), or ZnI_2_·2H_2_O (89 mg) were dissolved in acetone (4 mL), followed by the addition of 0.5 mmol of 2-ClPy (47 μL), 2-BrPy (48 μL) or 2-IPy (54 μL), and 1 mL of toluene. Slow evaporation of solvent resulted in formation of corresponding single crystals (colorless in all cases) suitable for XRD. Alternatively, 4 mL of ethanol could be used instead of acetone/toluene mixture (there form pure complexes, but the quality of crystals is lower). In all cases, yields were in the 89–94% range. The results of the element analysis for **1**–**9** are given in Supplementary Materials (Table S1).

### 3.2. X-ray Diffractometry

Data sets for single crystals of **1**–**7** were obtained at 130 (**1** and **2**), 140 (**3**–**5**), 150 (**6**), or 143 (**7**) K on Agilent Xcalibur diffractometer equipped with an area AtlasS2 detector (graphite monochromator, λ(MoKα) = 0.71073 Å, ω-scans). Integration, absorption correction, and determination of unit cell parameters were performed using the CrysAlisPro program package (CrysAlisPro 1.171.38.41. Rigaku Oxford Diffraction: the Woodlands, TX, USA, 2015). For the crystal of **9**, the data were obtained on Bruker D8 Venture diffractometer with a CMOS PHOTON III detector and IµS 3.0 source (Mo Kα radiation, λ = 0.71073 Å). All measurements were performed at 150 K, the φ- and ω-scan techniques were employed. Absorption correction was performed using the SADABS program (Bruker Apex3 software suite: Apex3, SADABS-2016/2 and SAINT, version 2018.7-2; Bruker AXS Inc.: Madison, WI, USA, 2017). The structures were solved by a dual space algorithm (SHELXT) and refined by the full-matrix least squares technique (SHELXL) [62] in the anisotropic approximation (except hydrogen atoms). Positions of hydrogen atoms of organic ligands were calculated geometrically and refined in the riding model. The crystallographic data and details of the structure refinements are summarized in Table S2 (Supplementary Information). CCDC 2059579-2059586 contain the supplementary crystallographic data for this paper. These data can be obtained free of charge from the Cambridge Crystallographic Data Center at http://www.ccdc.cam.ac.uk/data_request/cif. 

### 3.3. Computational Details

The single point calculations based on the experimental X-ray geometries of **1–7** and **9** were carried out at the DFT level of theory using the dispersion-corrected hybrid functional ωB97XD [63] with the help of the Gaussian-09 program package. The second-order scalar relativistic Douglas–Kroll–Hess calculations, requested relativistic core Hamiltonian, were carried out using the DZP-DKH basis sets [64,65,66,67] for all atoms. The topological analysis of the electron density distribution, with the help of the atoms in molecules (QTAIM) method developed by Bader [68], was performed by using the Multiwfn program (version 3.7) [69]. The Cartesian atomic coordinates for model supramolecular trimeric associates are presented in Table S3. Currently, two general theoretical approaches for studies of non-covalent interactions in the solid state are widespread. The first is the “molecular” approach that is typically applied for *molecular* crystals, and it includes modeling of a separate isolated supramolecular adduct without considering the neighboring molecular environment, and periodicity of the real crystal (for reviews see [70] and [71]). This is a rather rough approximation, but it is useful when fine effects are not under study and high accuracy is not needed. The second approach typically includes time-demanding “true” periodic conditions calculations. It is perfectly correct for highly symmetrical *ionic* crystals (for review see [72]), but has certain limitations for less symmetric, disordered molecular crystals, and crystallosolvates. In our recent works [73,74], we showed that a single point “molecular” approach for calculation of supramolecular associates, particularly involving transition metal complexes, agree well with Kohn–Sham calculations, with periodic boundary conditions. Moreover, other researchers widely used such single point “molecular” approaches in studies on halogen bonding and other non-covalent interactions in similar chemical systems [52,55,56].

### 3.4. Powder X-ray Diffractometry (PXRD)

Details are provided in Supplementary Material.

### 3.5. Luminescence Spectra

Registration of emission and excitation spectra at room temperature, as well as determination of absolute emission quantum yields, were performed with Fluorolog-3 (Horiba Jobin Yvon), equipped with a 450 W Xe lamp, an integration sphere, double grating excitation, and emission monochromators. Emission and excitation spectra were corrected for source intensity (lamp and grating) and emission spectral response (detector and grating) by standard correction curves. Lifetime measurements were performed on a Horiba FluoroCube lifetime instrument by a time-correlated single-photon counting method using a 350 nm LED excitation source.

## 4. Conclusions

Despite overall similarity of XB patterns in the crystal structures of [(2-XL)_2_ZnY_2_] complexes, not all members of this group are isostructural. The presence of halogen···halogen non-covalent interactions was detected by means of QTAIM analysis, even in cases where corresponding distances exceeded the sum of the van der Waals radii. Considering that similar situations were reported in previous studies, it can be concluded that judging on presence (or absence) of non-covalent interactions, based *only* on analysis of distances, can be misleading: in some “borderline” cases, DFT calculations give more reliable answers. Although most [(2-XL)_2_ZnY_2_] reveal moderate luminescence, there are two compounds demonstrating quantum yields exceeding 20%. Taking into account that XB can affect photophysical characteristics [2], examination of luminescent behavior of **1**–**9** in presence of other XB-forming building blocks (i.e., perfluoroiodoarenes) can be an interesting research task; corresponding experiments are underway in our group.

## Figures and Tables

**Table 1:** Selected geometric parameters (Å and °) in **1**–**9**. Data for **8** are omitted (see comments in main text for details).

1	Zn-Y	d_XY_	S_XY_	S_XY_-d_XY_	C-X-Y	Zn-Y-X
2	2.221–2.234	3.515; 3.541	3.50	−0.015, −0.041	165.0	122.4
3	2.221–2.235	3.421; 3.429	3.58	0.159, 0.151	164.1	129.2
4	2.234–2.241	3.414; 3.426	3.73	0.316, 0.304	162.9	128.8
5	2.360–2.372	3.562; 3.646	3.58	0.018, −0.066	166.5	121.9
6	2.366–2.379	3.498; 3.545	3.66	0.162, 0.115	166.4	122.3
7	2.375–2.389	3.530; 3.540	3.81	0.280, 0.270	162.5	125.5
9	2.566–2.580	3.757; 3.843	3.73	−0.027, −0.113	169.1	124.1
	2.585–2.591	3.693; 3.729	3.96	0.267, 0.231	162.5; 165.8	119.4, 123.7

**Table 2:** Isostructural complexes of [(2-XL)_2_MY_2_] series.

M	L	Y	Isostructural To	Reference
Sn(IV)	2-IPh	Cl	**1**–**7**	[44]
Sn(IV)	2-IPh	I	**8**–**9**	[44]
Co(II)	2-ClPy	Cl	**1**–**7**	[41]
Co(II)	2-BrPy	Cl	**1**–**7**	[41]
Co(II)	2-IPy	Cl	**1**–**7**	[41]
Co(II)	2-BrPy	Br	**1**–**7**	[45]
Zn(II)	2-MePy	Cl	**1**–**7**	[46]
Zn(II)	2-MePy	Br	**1**–**7**	[46]
Zn(II)	2-MePy	I	**-**	[46]
Cd(II)	2-MePy	Br	**1**–**7**	[46]
Cd(II)	2-MePy	I	**-**	[46]
Mg(II)	2-MePy	Br	**1**–**7**	[47]

**Table 3:** Values of the density of all electrons—ρ(**r**), Laplacian of electron density—∇^2^ρ(**r**) and appropriate λ_2_ eigenvalues, energy density—H_b_, potential energy density—V(**r**), and Lagrangian kinetic energy—G(**r**) (a.u.) at the bond critical points (3, −1), corresponding to the non-covalent interactions X···Y (X, Y = Cl, Br, I) in **1**–**7** and **9**, as well as energies for these contacts E_int_ (kcal/mol), defined by different approaches.

Contact, Å	ρ(r)	∇^2^ρ(r)	λ_2_	H_b_	V(r)	G(r)	E_int_^a^	E_int_^b^	E_int_^c^	E_int_^d^
**1**										
Cl···Cl, 3.515	0.006	0.022	−0.006	0.001	−0.003	0.004	0.9	1.1	0.9	1.2
Cl···Cl, 3.541	0.006	0.021	−0.006	0.001	−0.003	0.004	0.9	1.1	0.9	1.2
**2**										
Br···Cl, 3.421	0.009	0.029	−0.009	0.001	−0.005	0.006	1.6	1.6	1.8	2.1
Br···Cl, 3.428	0.009	0.029	−0.009	0.001	−0.005	0.006	1.6	1.6	1.8	2.1
**3**										
I···Cl, 3.414	0.012	0.040	−0.012	0.001	−0.008	0.009	2.5	2.4	3.4	3.8
I···Cl, 3.426	0.012	0.039	−0.012	0.002	−0.007	0.009	2.2	2.4	3.0	3.8
**4**										
Cl···Br, 3.561	0.007	0.023	−0.007	0.001	−0.004	0.005	1.3	1.3	1.2	1.5
Cl···Br, 3.647	0.006	0.019	−0.006	0.001	−0.003	0.004	0.9	1.1	0.9	1.2
**5**										
Br···Br, 3.498	0.010	0.027	−0.010	0.001	−0.005	0.006	1.6	1.6	1.8	2.1
Br···Br, 3.545	0.009	0.024	−0.009	0.000	−0.005	0.005	1.6	1.3	1.8	1.8
**6**										
I···Br, 3.530	0.012	0.034	−0.012	0.001	−0.007	0.008	2.2	2.2	3.0	3.4
I···Br, 3.540	0.012	0.034	−0.012	0.001	−0.007	0.008	2.2	2.2	3.0	3.4
**7**										
Cl···I, 3.758	0.006	0.022	−0.006	0.001	−0.003	0.004	0.9	1.1	0.9	1.2
Cl···I, 3.843	0.005	0.019	−0.005	0.002	−0.002	0.004	0.6	1.1	0.6	1.2
**9**										
I···I, 3.693	0.011	0.037	−0.011	0.001	−0.007	0.008	2.2	2.2	3.0	3.4
I···I, 3.729	0.010	0.036	−0.010	0.001	−0.007	0.008	2.2	2.2	3.0	3.4

^a^E_int_ = −V(**r**)/2 for all types of non-covalent interactions [58] ^b^ E_int_ = 0.429G(**r**) for all types of non-covalent interactions [59] ^c^ E_int_ = 0.49(−V(**r**)) or 0.58(−V(**r**)) or 0.68(−V(**r**)) for non-covalent interactions involving chlorine, bromine, and iodine atoms as halogen bond donor, respectively, [60], ^d^ E_int_ = 0.47G(**r**) or 0.57G(**r**) or 0.67G(**r**) for non-covalent interactions involving chlorine, bromine, and iodine atoms as a halogen bond donor, respectively [60].

**Table 4:** Isostructural complexes of [(2-XL)_2_MY_2_] series.

Compound	λem, nm	Quantum Yields (QY), %	Decay Lifetime τ, ns
**1**	360	21.4 (λ_ex_ = 310 nm)	4.8
	430	8.7 (λ_ex_ = 350 nm)	8.2
**2**	380	0.2 (λ_ex_ = 320 nm)	-
	500	1.4 (λ_ex_ = 400 nm)	-
**3**	400	-	-
	500	-	-
**4**	360	7.7 (λ_ex_ = 300 nm)	1.9
	420	-	-
	455	20 (λ_ex_ = 350 nm)	7.9
**5**	360	-	-
	450	-	-
**6**	400	-	-
	450	-	-
**7**	510	2.7 (λ_ex_ = 330 nm)	0.7
	640	3.5 (λ_ex_ = 430 nm)	0.2
**8**	No emission		
**9**	520	-	-
	580	0.8 (λ_ex_ = 460 nm)	-
**10**	350	1.6 (λ_ex_ = 300 nm)	0.9
	460	2.1 (λ_ex_ = 370 nm)	0.4
**11**	430	1.2 (λ_ex_ = 340 nm)	0.9
	520	1.1 (λ_ex_ = 400 nm)	0.4
**12**	No emission		

## Data Availability

Not applicable.

## Supplementary Materials

The following are available online: XRD and PXRD data, luminescence spectra and details of DFT calculations.

## Sample Availability

Samples of the compounds are available from the authors.

## References

[1] Desiraju G.R., Ho P.S., Kloo L., Legon A.C., Marquardt R., Metrangolo P., Politzer P., Resnati G., Rissanen K. (2013). Definition of the halogen bond (IUPAC Recommendations 2013). Pure Appl. Chem..

[2] Sivchik V.V., Solomatina A.I., Chen Y.-T., Karttunen A.J., Tunik S.P., Chou P.-T., Koshevoy I.O. (2015). Halogen Bonding to Amplify Luminescence: A Case Study Using a Platinum Cyclometalated Complex. Angew. Chem. Int. Ed..

[3] Xiao L., Fu H. (2019). Enhanced Room-Temperature Phosphorescence through Intermolecular Halogen/Hydrogen Bonding. Chem. A Eur. J..

[4] Cai S., Shi H., Tian D., Ma H., Cheng Z., Wu Q., Gu M., Huang L., An Z., Peng Q. (2018). Enhancing Ultralong Organic Phosphorescence by Effective π-Type Halogen Bonding. Adv. Funct. Mater..

[5] Sivchik V., Sarker R.K., Liu Z.-Y., Chung K.-Y., Grachova E.V., Karttunen A.J., Chou P.-T., Koshevoy I.O. (2018). Improvement of the Photophysical Performance of Platinum-Cyclometalated Complexes in Halogen-Bonded Adducts. Chem. A Eur. J..

[6] Chowdhury B., Sinha S., Ghosh P. (2016). Selective Sensing of Phosphates by a New Bis-heteroleptic Ru^II^Complex through Halogen Bonding: A Superior Sensor over Its Hydrogen-Bonding Analogue. Chem. A Eur. J..

[7] Sun J., Riel A.M.S., Berryman O.B. (2018). Solvatochromism and fluorescence response of a halogen bonding anion receptor. New J. Chem..

[8] Ponnusamy K., Chellappan S., Singaravelu C.M., Kandasamy J. (2018). Anion halogen bonding effect on solvatochromism and excited state dynamics of hemicyanine dye in chlorinated solvents. J. Lumin..

[9] Yang H., Wong M.W. (2020). Application of halogen bonding to organocatalysis: A theoretical perspective. Molecules.

[10] Szell P.M.J., Zablotny S., Bryce D.L. (2019). Halogen bonding as a supramolecular dynamics catalyst. Nat. Commun..

[11] Sutar R.L., Huber S.M. (2019). Catalysis of Organic Reactions through Halogen Bonding. ACS Catal..

[12] Mikherdov A.S., Novikov A.S., Boyarskiy V.P., Kukushkin V.Y. (2020). The halogen bond with isocyano carbon reduces isocyanide odor. Nat. Commun..

[13] Mullaney B.R., Thompson A.L., Beer P.D. (2014). An All-Halogen Bonding Rotaxane for Selective Sensing of Halides in Aqueous Media. Angew. Chem. Int. Ed..

[14] Jaini A.K.A., Hughes L.B., Kitimet M.M., Ulep K.J., Leopold M.C., Parish C.A. (2019). Halogen bonding interactions for aromatic and nonaromatic explosive detection. ACS Sens..

[15] Weis J.G., Ravnsbæk J.B., Mirica K.A., Swager T.M. (2016). Employing Halogen Bonding Interactions in Chemiresistive Gas Sensors. ACS Sens..

[16] Vitorica-Yrezabal I.J., Sullivan R.A., Purver S.L., Curfs C., Tang C.C., Brammer L. (2011). Synthesis and polymorphism of (4-ClpyH)_2_[CuCl_4_]: Solid–gas and solid–solid reactions. CrystEngComm.

[17] Zordan F., Brammer L. (2006). M−X···X′−C Halogen-Bonded Network Formation in MX_2_ (4-halopyridine)_2_ Complexes (M = Pd, Pt; X = Cl, I.; X′ = Cl, Br, I). Cryst. Growth Des..

[18] Zordan F., Brammer L., Sherwood P. (2005). Supramolecular Chemistry of Halogens: Complementary Features of Inorganic (M−X) and Organic (C−X‘) Halogens Applied to M−X···X‘−C Halogen Bond Formation. J. Am. Chem. Soc..

[19] Mínguez Espallargas G., van de Streek J., Fernandes P., Florence A.J., Brunelli M., Shankland K., Brammer L. (2010). Mechanistic Insights into a Gas-Solid Reaction in Molecular Crystals: The Role of Hydrogen Bonding. Angew. Chem. Int. Ed..

[20] Mínguez Espallargas G., Florence A.J., van de Streek J., Brammer L. (2011). Different structural destinations: Comparing reactions of [CuBr_2_(3-Brpy)_2_] crystals with HBr and HCl gas. CrystEngComm.

[21] Clemente-Juan J.M., Coronado E., Mínguez Espallargas G., Adams H., Brammer L. (2010). Effects of halogen bonding in ferromagnetic chains based on Co(II) coordination polymers. CrystEngComm.

[22] Krasinski C.A., Solomon B.L., Awwadi F.F., Landee C.P., Turnbull M.M., Wikaira J.L. (2017). Copper(II) halide salts and complexes of 4-amino-2-fluoropyridine: Synthesis, structure and magnetic properties. J. Coord. Chem..

[23] Awwadi F.F., Turnbull M.M., Alwahsh M.I., Haddad S.F. (2018). May halogen bonding interactions compete with Cu⋯Cl semi-coordinate bonds? Structural, magnetic and theoretical studies of two polymorphs of *trans*-bis(5-bromo-2-chloropyridine)dichlorocopper(II) and *trans*-bis(2,5-dichloropyridine)dichlorocopper(II). New J. Chem..

[24] Awwadi F.F., Willett R.D., Twamley B., Turnbull M.M., Landee C.P. (2015). Dual Behavior of Bromine Atoms in Supramolecular Chemistry: The Crystal Structure and Magnetic Properties of Two Copper(II) Coordination Polymers. Cryst. Growth Des..

[25] Awwadi F., Willett R.D., Twamley B. (2011). Tuning Molecular Structures Using Weak Noncovalent Interactions: Theoretical Study and Structure of *trans* -Bis(2-chloropyridine)dihalocopper(II) and *trans* -Bis(3-chloropyridine)dibromocopper(II). Cryst. Growth Des..

[26] Awwadi F.F., Willett R.D., Haddad S.F., Twamley B. (2006). The electrostatic nature of aryl-bromine-halide synthons: The role of aryl-bromine-halide synthons in the crystal structures of the trans-bis(2-bromopyridine)dihalocopper(II) and trans-bis(3-bromopyridine)dihalocopper(II) complexes. Cryst. Growth Des..

[27] Puttreddy R., von Essen C., Rissanen K. (2018). Halogen Bonds in Square Planar 2,5-Dihalopyridine-Copper(II) Bromide Complexes. Eur. J. Inorg. Chem..

[28] Shortsleeves K.C., Dawe L.N., Landee C.P., Turnbull M.M. (2009). Transition metal complexes of 2-amino-3,5-dihalopyridines: Synthesis, structures and magnetic properties of Cu(3,5-diCAP)_2_X_2_ and Cu(3,5-diBAP)_2_X_2_. Inorg. Chim. Acta.

[29] Puttreddy R., von Essen C., Peuronen A., Lahtinen M., Rissanen K. (2018). Halogen bonds in 2,5-dihalopyridine-copper(II) chloride complexes. CrystEngComm.

[30] Farris P.C., Wall A.D., Chellali J.E., Chittim C.L., Landee C.P., Turnbull M.M., Wikaira J.L. (2018). Copper(II) halide complexes of aminopyridines: Syntheses, structures and magnetic properties of [(5CAP)_2_CuX_2_] and [(5BAP)_n_CuX_2_] (X = Cl, Br). J. Coord. Chem..

[31] Dubois R.J., Landee C.P., Rademeyer M., Turnbull M.M. (2019). Pyridine-based complexes of copper(II) chloride and bromide: Ligand conformation effects on crystal structure. Synthesis, structure and magnetic behavior of Cu(2-Cl-3-X′py)_2_X_2_ [X, X′ = Cl, Br]. J. Coord. Chem..

[32] Hu C., Kalf I., Englert U. (2007). Pyridine complexes of mercury(ii)halides: Implications of a soft metal center for crystal engineering. CrystEngComm.

[33] Wang R., Dols T.S., Lehmann C.W., Englert U. (2012). The halogen bond made visible: Experimental charge density of a very short intermolecular Cl⋯Cl donor–acceptor contact. Chem. Commun..

[34] Hu C., Li Q., Englert U. (2003). Structural trends in one and two dimensional coordination polymers of cadmium(II) with halide bridges and pyridine-type ligands. CrystEngComm.

[35] Awwadi F.F., Haddad S.F., Turnbull M.M., Landee C.P., Willett R.D. (2013). Copper–halide bonds as magnetic tunnels; structural, magnetic and theoretical studies of trans-bis(2,5-dibromopyridine)dihalocopper(II) and trans-bis(2-bromopyridine)dibromocopper(II). CrystEngComm.

[36] Hu C., Englert U. (2001). Polymeric versus monomeric and tetrahedral versus octahedral coordination in zinc(II) pyridine complexes. CrystEngComm.

[37] Małecki J.G., Maroń A. (2017). Luminescence properties of copper(I), zinc(II) and cadmium(II) coordination compounds with picoline ligands. J. Lumin..

[38] Espallargas G.M., Brammer L., van de Streek J., Shankland K., Florence A.J., Adams H. (2006). Reversible Extrusion and Uptake of HCl Molecules by Crystalline Solids Involving Coordination Bond Cleavage and Formation. J. Am. Chem. Soc..

[39] Bondi A. (1966). Van der Waals Volumes and Radii of Metals in Covalent Compounds. J. Phys. Chem..

[40] Mantina M., Chamberlin A.C., Valero R., Cramer C.J., Truhlar D.G. (2009). Consistent van der Waals Radii for the Whole Main Group. J. Phys. Chem. A.

[41] Adonin S.A., Bondarenko M.A., Novikov A.S., Sokolov M.N. (2020). Halogen Bonding in Isostructural Co(II) Complexes with 2-Halopyridines. Crystals.

[42] Ivanov D.M., Novikov A.S., Starova G.L., Haukka M., Kukushkin V.Y. (2016). A family of heterotetrameric clusters of chloride species and halomethanes held by two halogen and two hydrogen bonds. CrystEngComm.

[43] Kryukova M.A., Ivanov D.M., Kinzhalov M.A., Novikov A.S., Smirnov A.S., Bokach N.A., Kukushkin V.Y. (2019). Four-Center Nodes: Supramolecular Synthons Based on Cyclic Halogen Bonding. Chem. A Eur. J..

[44] Leonhardt T., Latscha H.P. (1997). Photochemical Synthesis of New Tinorganic Compounds with Active Substituents on Tin|Photochemische Synthese neuer zinnorganischer Verbindungen mit “aktiven” Gruppen am Zinnatom (I). Z. Naturforsch. Sect. B J. Chem. Sci..

[45] Hiltunen L., Niinistö L., Kenessey G., Keserü G.M., Liptay G., Kondow A.J., Bisht K.S., Parmar V.S., Francis G.W. (1994). Pyridine-Type Complexes of Transition-Metal Halides. V. Preparation, Thermal Properties, Infrared Spectra and Crystal Structure of Dibromo-bis(2-bromopyridine)cobalt(II). Acta Chem. Scand..

[46] Bowmaker G.A., Effendy F., Rahajoe S.I., Skelton B.W., White A.H. (2011). Structural and infrared spectroscopic studies of some adducts of divalent metal dihalides (MX_2_, M = Zn, Cd; X = CI, Br, I) with variously hindered monodentate nitrogen (pyridine) base ligands (L = pyridine, 2-methylpyridine, and quinoline) of 1:2 Stoichiometry. Z. Anorg. Allg. Chem..

[47] Waters A.F., White A.H. (1996). Synthesis and structural systematics of nitrogen base adducts of group 2 salts. II: Some adducts of group 2 salts with pyridine. Aust. J. Chem..

[48] Ding X., Tuikka M.J., Hirva P., Kukushkin V.Y., Novikov A.S., Haukka M. (2016). Fine-tuning halogen bonding properties of diiodine through halogen–halogen charge transfer extended [Ru(2,2′-bipyridine)(CO)_2_X_2_]·I_2_ systems (X = Cl, Br, I). CrystEngComm.

[49] Mikherdov A.S., Kinzhalov M.A., Novikov A.S., Boyarskiy V.P., Boyarskaya I.A., Dar’in D.V., Starova G.L., Kukushkin V.Y. (2016). Difference in Energy between Two Distinct Types of Chalcogen Bonds Drives Regioisomerization of Binuclear (Diaminocarbene)Pd^II^ Complexes. J. Am. Chem. Soc..

[50] Bulatova M., Melekhova A.A., Novikov A.S., Ivanov D.M., Bokach N.A. (2018). Redox reactive (RNC)Cu^II^ species stabilized in the solid state via halogen bond with I_2_. Z. Krist. Cryst. Mater..

[51] Kashina M.V., Kinzhalov M.A., Smirnov A.S., Ivanov D.M., Novikov A.S., Kukushkin V.Y. (2019). Dihalomethanes as Bent Bifunctional XB/XB-Donating Building Blocks for Construction of Metal-involving Halogen Bonded Hexagons. Chem. Asian J..

[52] Sharma P., Gogoi A., Verma A.K., Frontera A., Bhattacharyya M.K. (2020). Charge-assisted hydrogen bond and nitrile···nitrile interaction directed supramolecular associations in Cu(II) and Mn(II) coordination complexes: Anticancer, hematotoxicity and theoretical studies. New J. Chem..

[53] Kar P., Franconetti A., Frontera A., Ghosh A. (2019). Chloranilate bridged dinuclear copper(II) complexes: Syn Anti geometry tuned by the steric factor and supramolecular interactions. CrystEngComm.

[54] Banerjee A., Frontera A., Chattopadhyay S. (2019). Methylene spacer regulated variation in molecular and crystalline architectures of cobalt(III) complexes with reduced Schiff base ligands: A combined experimental and theoretical study. Dalt. Trans..

[55] Basak T., Frontera A., Chattopadhyay S. (2021). Insight into non-covalent interactions in two triamine-based mononuclear iron(III) Schiff base complexes with special emphasis on the formation of Br···π halogen bonding. CrystEngComm.

[56] Hajiashrafi T., Salehi S., Kubicki M., Bauzá A., Frontera A., Flanagan K.J., Senge M.O. (2019). Solid-state supramolecular architectures of a series of Hg(II) halide coordination compounds based on hydroxyl-substituted Schiff base ligands. CrystEngComm.

[57] Mirzaei M., Eshtiagh-Hosseini H., Bolouri Z., Rahmati Z., Esmaeilzadeh A., Hassanpoor A., Bauza A., Ballester P., Barceló-Oliver M., Mague J.T. (2015). Rationalization of noncovalent interactions within six new M^II^/8-aminoquinoline supramolecular complexes (M^II^ = Mn, Cu, and Cd): A combined experimental and theoretical DFT study. Cryst. Growth Des..

[58] Espinosa E., Molins E., Lecomte C. (1998). Hydrogen bond strengths revealed by topological analyses of experimentally observed electron densities. Chem. Phys. Lett..

[59] Vener M.V., Egorova A.N., Churakov A.V., Tsirelson V.G. (2012). Intermolecular hydrogen bond energies in crystals evaluated using electron density properties: DFT computations with periodic boundary conditions. J. Comput. Chem..

[60] Bartashevich E.V., Tsirelson V.G. (2014). Interplay between non-covalent interactions in complexes and crystals with halogen bonds. Russ. Chem. Rev..

[61] Wang Y., Townsend P.D. (2013). Potential problems in collection and data processing of luminescence signals. J. Lumin..

[62] Sheldrick G.M. (2015). Crystal structure refinement with *SHELXL*. Acta Crystallogr. Sect. C Struct. Chem..

[63] Chai J.D., Head-Gordon M. (2008). Long-range corrected hybrid density functionals with damped atom-atom dispersion corrections. Phys. Chem. Chem. Phys..

[64] Barros C.L., de Oliveira P.J.P., Jorge F.E., Canal Neto A., Campos M. (2010). Gaussian basis set of double zeta quality for atoms Rb through Xe: Application in non-relativistic and relativistic calculations of atomic and molecular properties. Mol. Phys..

[65] Jorge F.E., Canal Neto A., Camiletti G.G., Machado S.F. (2009). Contracted Gaussian basis sets for Douglas–Kroll–Hess calculations: Estimating scalar relativistic effects of some atomic and molecular properties. J. Chem. Phys..

[66] Canal Neto A., Jorge F.E. (2013). All-electron double zeta basis sets for the most fifth-row atoms: Application in DFT spectroscopic constant calculations. Chem. Phys. Lett..

[67] de Berrêdo R.C., Jorge F.E. (2010). All-electron double zeta basis sets for platinum: Estimating scalar relativistic effects on platinum(II) anticancer drugs. J. Mol. Struct..

[68] Bader R.F.W. (1991). A quantum theory of molecular structure and its applications. Chem. Rev..

[69] Lu T., Chen F. (2012). Multiwfn: A multifunctional wavefunction analyzer. J. Comput. Chem..

[70] Řezáč J., Hobza P. (2016). Benchmark Calculations of Interaction Energies in Noncovalent Complexes and Their Applications. Chem. Rev..

[71] Kolář M.H., Hobza P. (2016). Computer Modeling of Halogen Bonds and Other σ-Hole Interactions. Chem. Rev..

[72] Beran G.J.O. (2016). Modeling Polymorphic Molecular Crystals with Electronic Structure Theory. Chem. Rev..

[73] Dabranskaya U., Ivanov D.M., Novikov A.S., Matveychuk Y.V., Bokach N.A., Kukushkin V.Y. (2019). Metal-Involving Bifurcated Halogen Bonding C–Br···η^2^ (Cl–Pt). Cryst. Growth Des..

[74] Ivanov D.M., Baykov S.V., Novikov A.S., Romanenko G., Bokach N.A., Evarestov R.A., KuKushkin V.Y. (2020). Noncovalent Sulfoxide-Nitrile Coupling Involving Four-Center Heteroleptic Dipole-Dipole Interactions between the Sulfinyl and Nitrile Groups. Cryst. Growth Des..

