# Supplementary material for: Zn(II) Heteroleptic Halide Complexes with 2-Halopyridines: Features of Halogen Bonding in Solid State
Source: Molecules. 2021 Jun 3;26(11):3393. doi: 10.3390/molecules26113393 (PMC8199927; doi:10.3390/molecules26113393)
Supplement: Supplementary file 1 [file molecules-26-03393-s001.zip › molecules-1214176-supplementary.pdf]

Zn(II) heteroleptic halide complexes with 2-halopyridines: features of halogen bonding in solid state

Mikhail A. Vershinin, Marianna I. Rakhmanova, Alexander S. Novikov, Maxim N. Sokolov and Sergey A. Adonin*

Powder X-ray diffractometry. XRD analysis of polycrystals was performed on Shimadzu XRD-7000 diffractometer (CuK-alpha radiation, Ni – filter, linear One Sight detector, 5 – 50° 2θ range, 0.0143° 2θ step, 2s per step). A polycrystalline sample was slightly ground with hexane in an agate mortar, and the resulting suspension was deposited on the polished side of a standard quartz sample holder, and a smooth thin layer being formed after drying. Plotting of PXRD patterns and data treatment was performed using X'Pert Plus software.

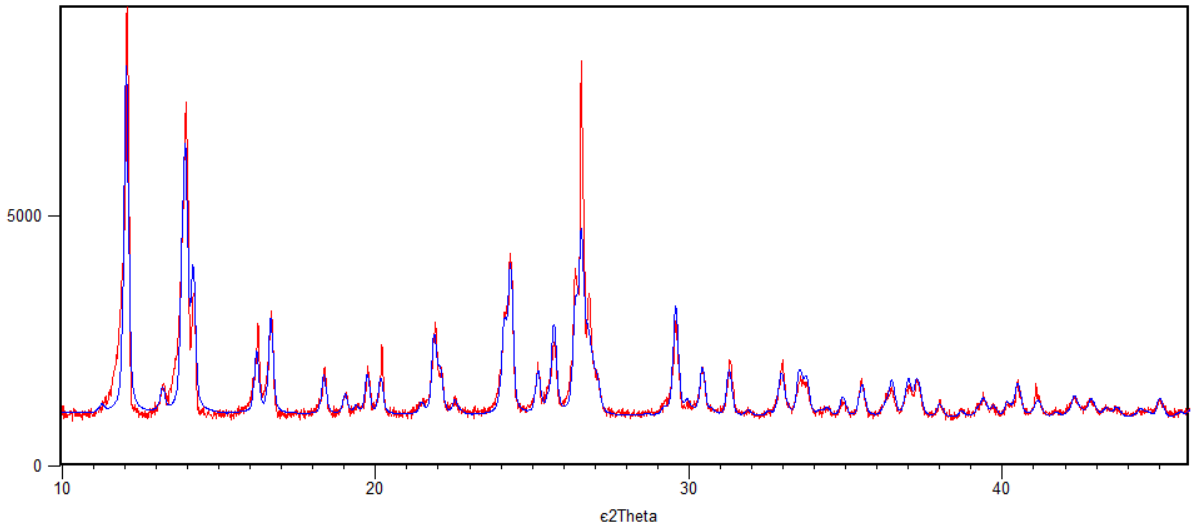

Figure S1. Experimental (blue) and calculated (red) PXRD patterns for **1**

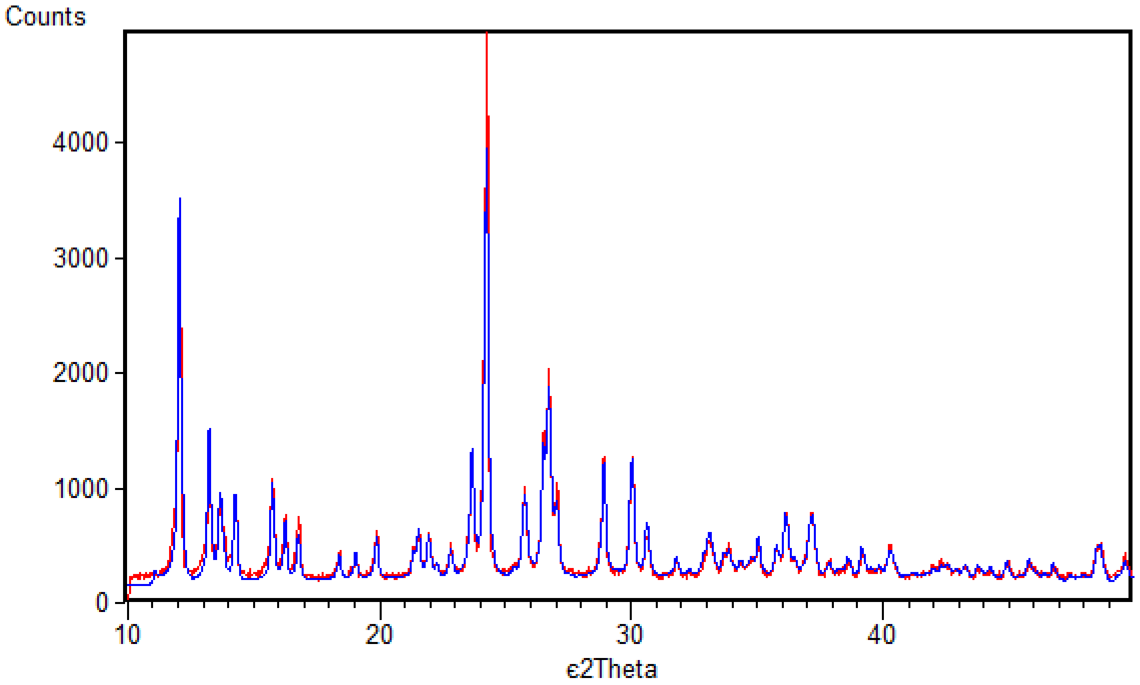

Figure S2. Experimental (blue) and calculated (red) PXRD patterns for **2**

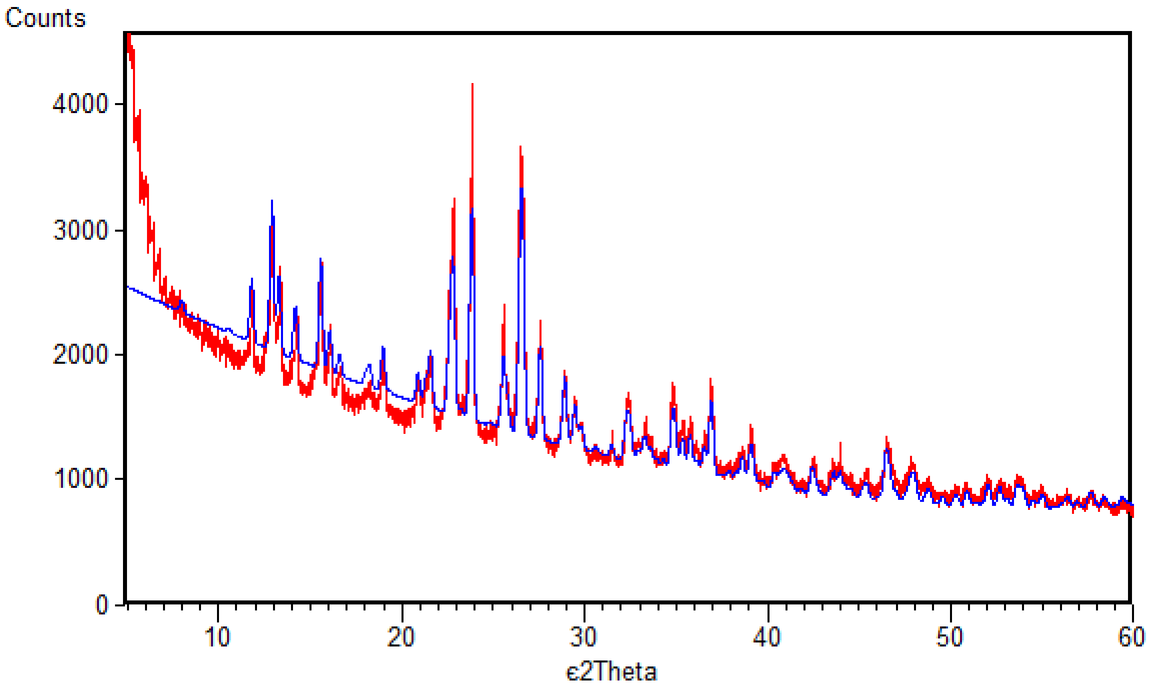

Figure S3. Experimental (blue) and calculated (red) PXRD patterns for **3**

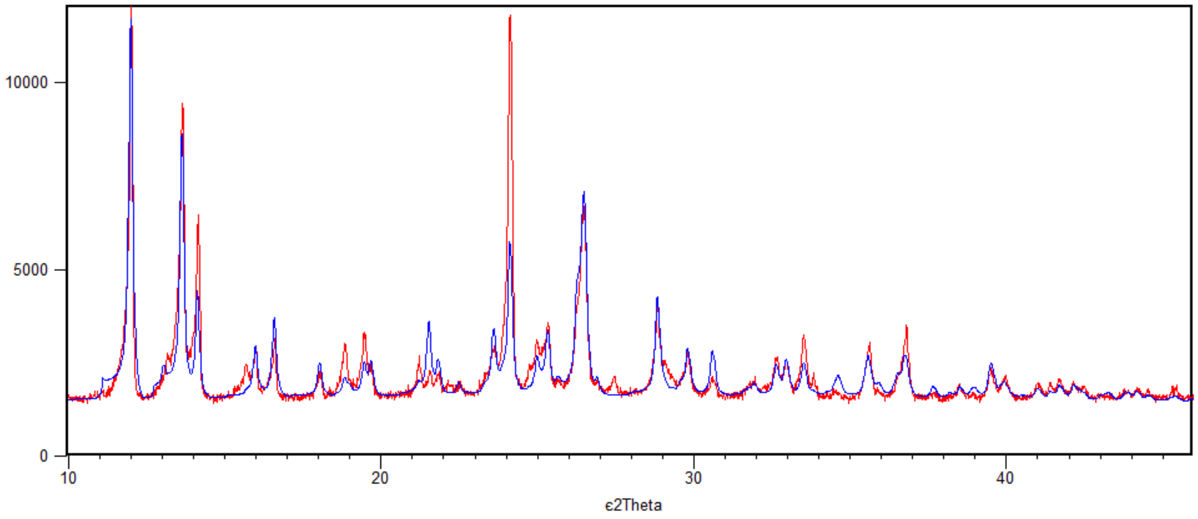

Figure S4. Experimental (blue) and calculated (red) PXRD patterns for **4**

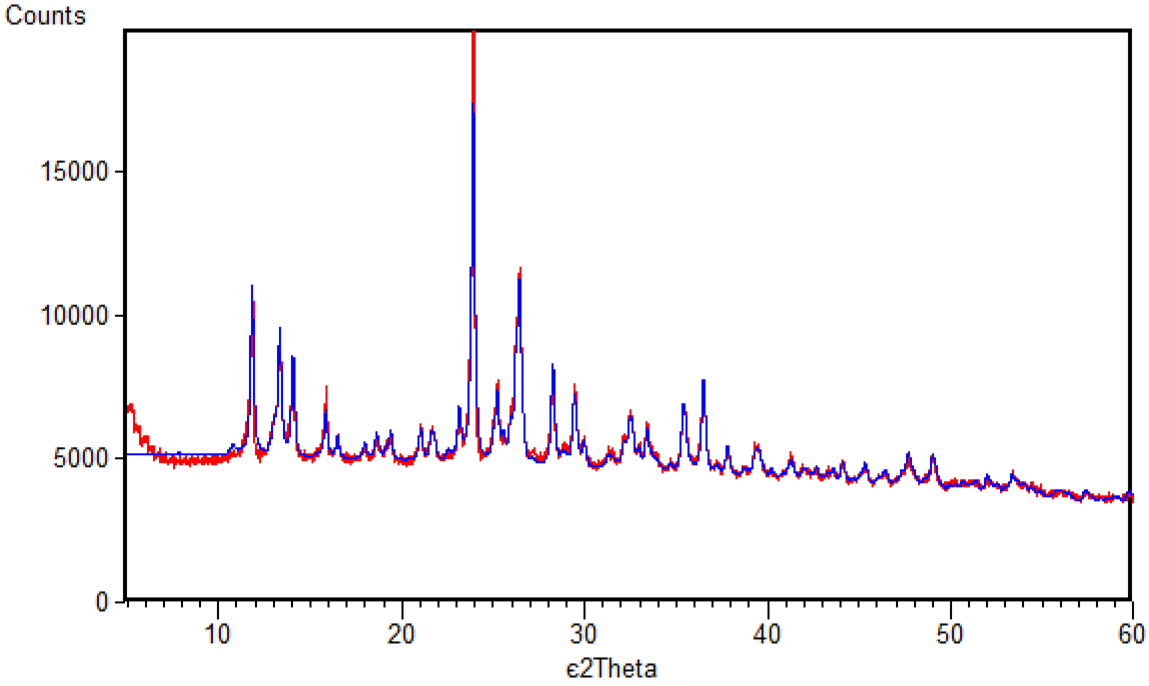

Figure S5. Experimental (blue) and calculated (red) PXRD patterns for **5**

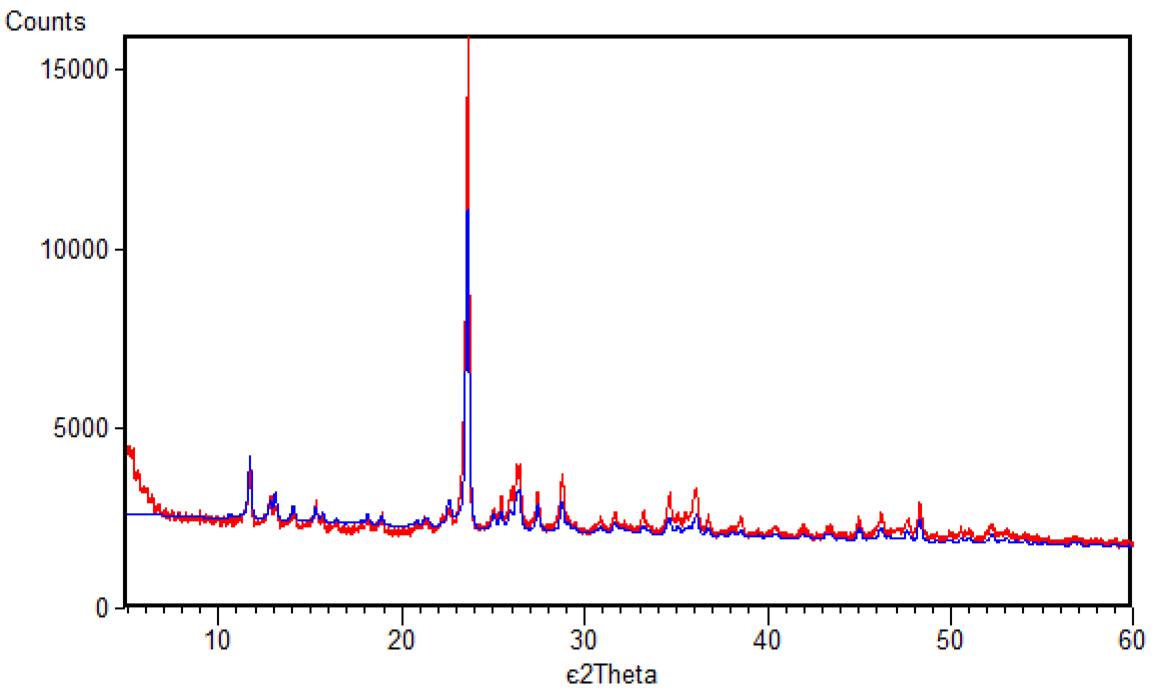

Figure S6. Experimental (blue) and calculated (red) PXRD patterns for **6**

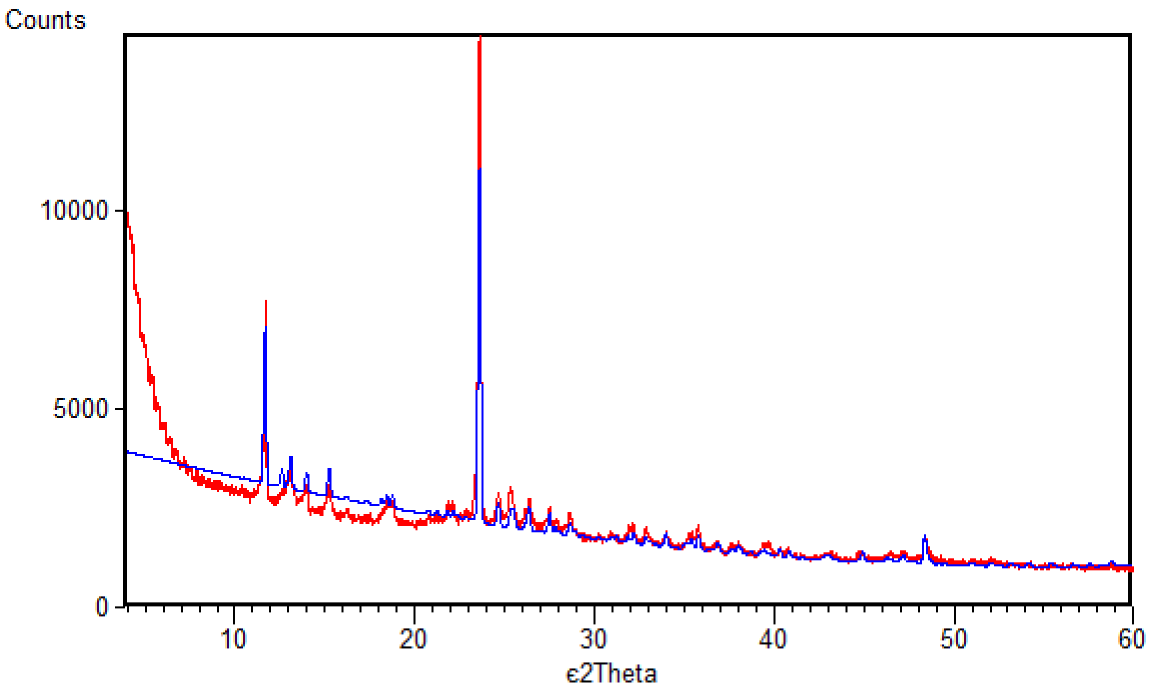

Figure S7. Experimental (blue) and calculated (red) PXRD patterns for **7**

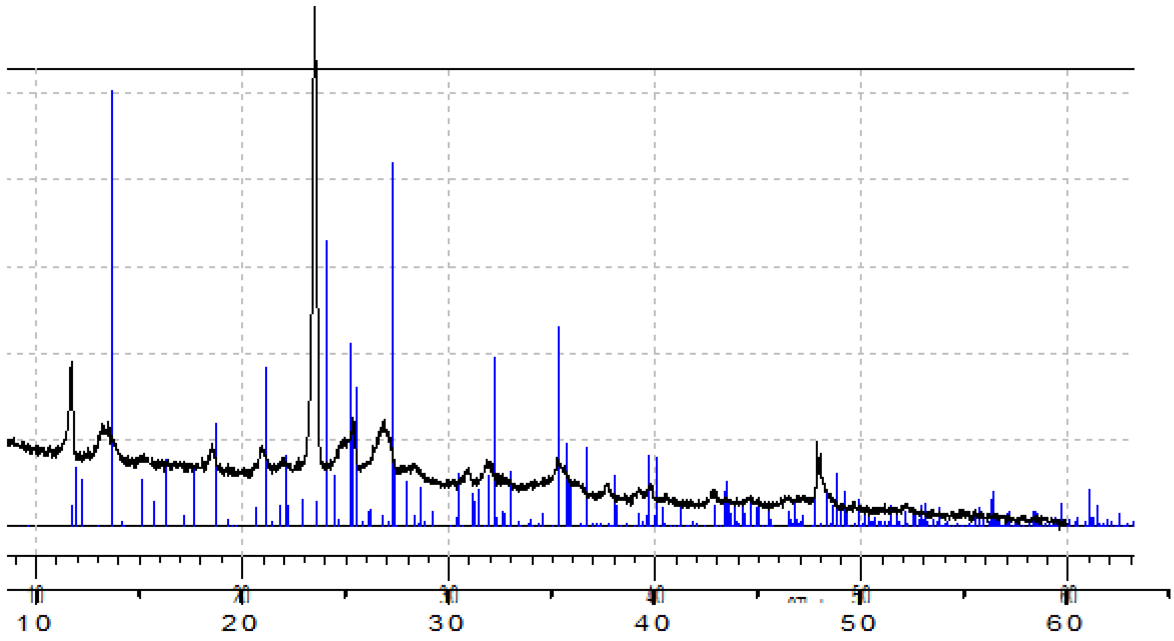

Figure S8. Experimental (black) and calculated (blue) PXRD patterns for **8** (calculation based on the structure of **9**)

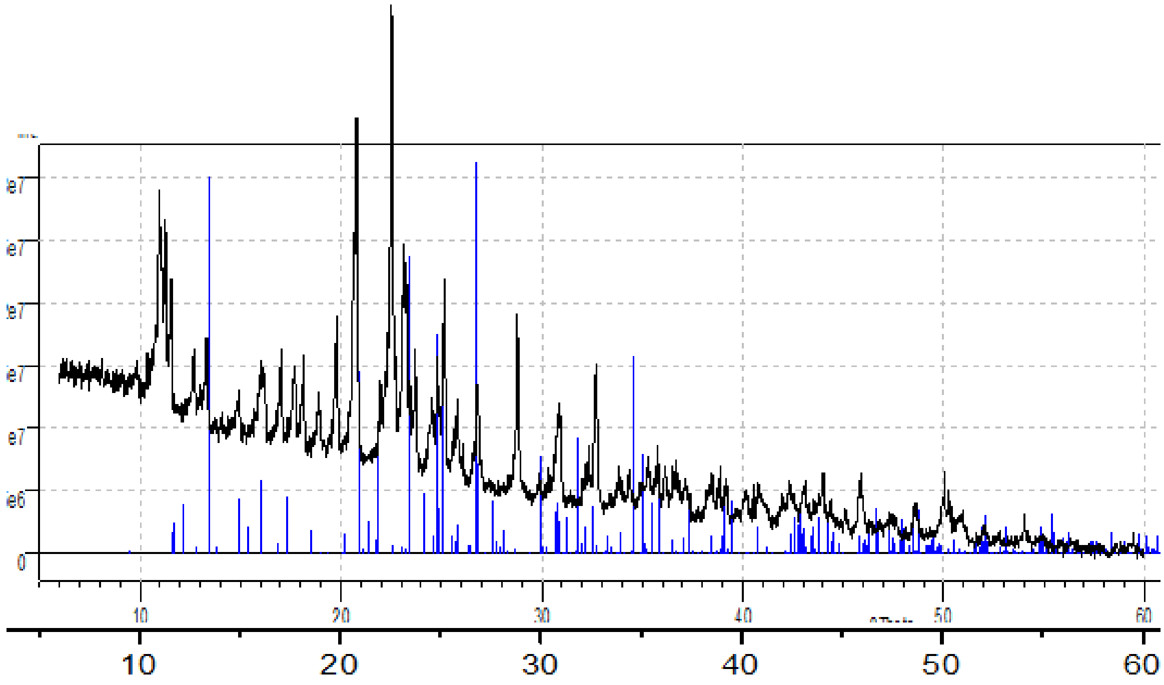

Figure S9. Experimental (black) and calculated (blue) PXRD patterns for **9**

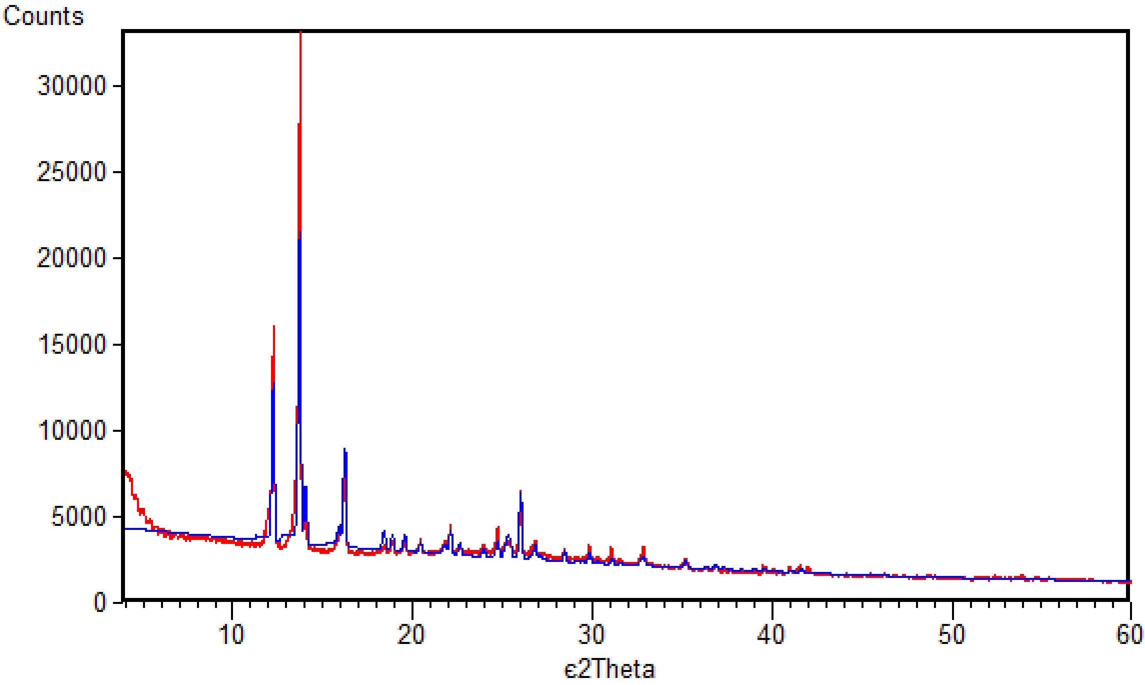

Figure S10. Experimental (blue) and calculated (red) PXRD patterns for **10**

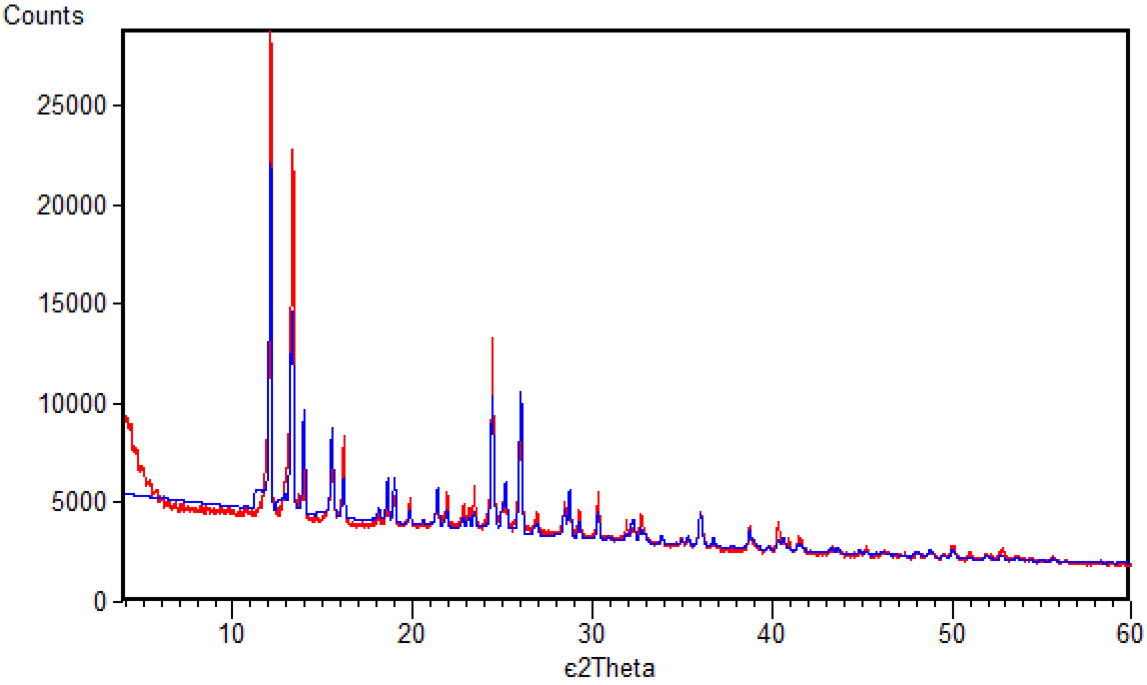

Figure S11. Experimental (blue) and calculated (red) PXRD patterns for **11**

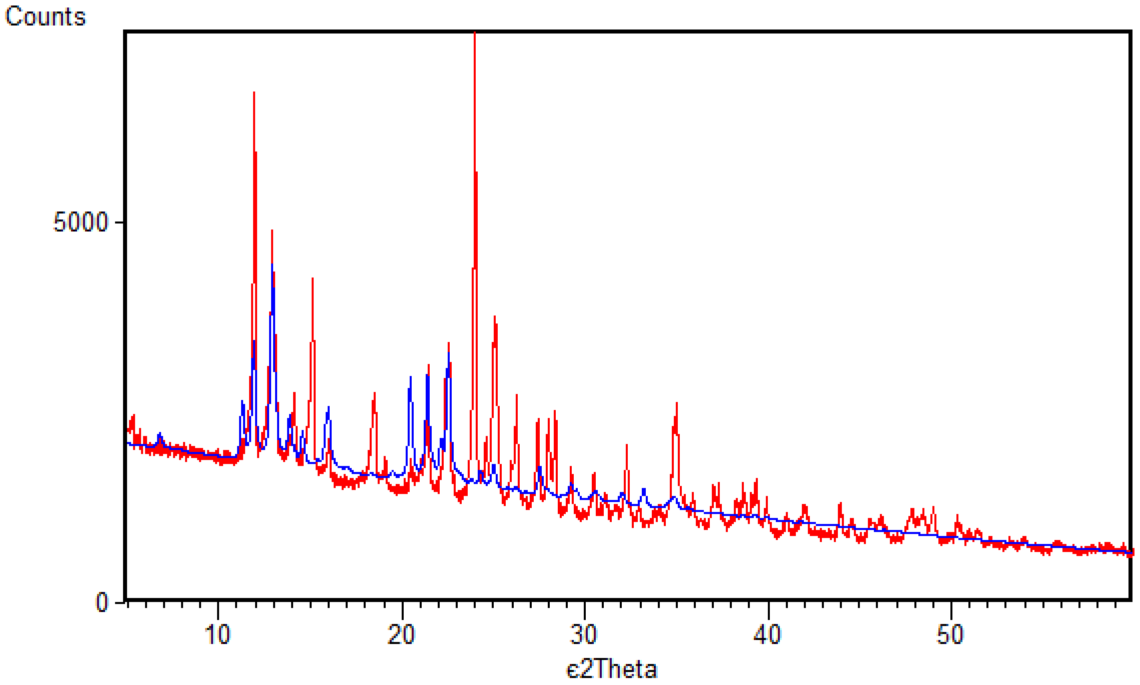

Figure S12. Experimental (blue) and calculated (red) PXRD patterns for **12**

Table 1S. Element analysis data for **1**-**9**

Compound	C, H, N, calcd	C, H, N, found
**1**	33.35, 2.24, 7.78	33.72; 2.29; 7.84
**2**	26.80, 1.80, 6.25	27.02; 1.84; 6.33
**3**	22.07; 1.48; 5.15	22.16; 1.57; 5.23
**4**	26.80, 1.80, 6.25	27.04; 1.87; 6.37
**5**	22.40; 1.51; 5.23	22.71; 1.58; 5.31
**6**	19.00; 1.28; 4.43	18.81; 1.32; 4.57
**7**	22.07; 1.48; 5.15	21,79; 1,46; 5.03
**8**	19.00; 1.28; 4.43	18.87; 1.29; 4.37
**9**	16.49; 1.11; 3.85	16.57; 1.12; 3.87

Table 2S. Crystal data and structure refinement for **1-7** and **9**

	**1**	**2**	**3**	**4**	**5**	**6**	**7**	**9**
Empirical formula	$C_{10}H_8Cl_4N_2Zn$	$C_{10}H_8Br_2Cl_2N_2Zn$	$C_{10}H_8Cl_2I_2N_2Zn$	$C_{10}H_8Br_2Cl_2N_2Zn$	$C_{10}H_8Br_4N_2Zn$	$C_{10}H_8Br_2I_2N_2Zn$	$C_{10}H_8Cl_2I_2N_2Zn$	$C_{10}H_8I_4N_2Zn$
M, g/mol	363.35	452.27	546.25	452.27	541.19	635.18	546.25	729.15
Crystal system, space group	Triclinic, *P*-1	Triclinic, *P*-1	Triclinic, *P*-1	Triclinic, *P*-1	Triclinic, *P*-1	Triclinic, *P*-1	Triclinic, *P*-1	Monoclinic, $P2_1/n$
a,b,c, Å	7.9423 (5), 8.3152 (5), 11.0341 (5)	7.9146 (5), 8.4277 (6), 11.0498 (6)	7.9452 (4), 8.6864 (4), 11.1868 (5)	7.9347 (4), 8.4869 (8), 11.2381 (10)	7.9547 (4), 8.5643 (5), 11.2938 (4)	8.0186 (5), 8.7153 (4), 11.4729 (7)	7.9153 (5), 8.7199 (5), 11.6868 (7)	8.8619 (4), 15.1663 (7), 11.8971 (6)
α,β,γ°	85.391 (4), 77.674 (4), 68.667 (5)	85.571 (5), 78.054 (5), 69.729 (6)	86.820 (4), 78.182 (4), 71.658 (5)	85.754 (7), 77.454 (6), 69.687 (6)	85.769 (4), 78.058 (4), 70.447 (5)	86.326 (4), 78.408 (5), 71.749 (5)	87.084 (4), 77.985 (5), 72.329 (5)	90, 92.969 (2), 90
V, Å^3	663.13 (7)	676.41 (8)	717.27 (6)	692.77 (10)	709.34 (6)	745.91 (8)	751.67 (8)	1596.85 (13)
Z	2	2	2	2	2	2	2	4
F(000)	360	432	504	432	504	576	504	1296
T_{min}, T_{max}	0.873, 1.000	0.534, 1.000	0.623, 1.000	0.497, 1.000	0.602, 1.000	0.585, 1.000	0.871, 1.000	0.454, 0.746

No. of measured, independent and observed [$I > 2\sigma(I)$] reflections	4886, 2908, 2468	4637, 2585, 2212	5309, 2717, 2380	4920, 2631, 2129	5098, 2693, 2352	5424, 2802, 2352	5427, 2854, 2376	27006, 2952, 2890
R_{int}	0.026	0.029	0.026	0.026	0.026	0.034	0.032	0.037
θ values (°)	θ_{max} = 28.9, θ_{min} = 1.9	θ_{max} = 25.7, θ_{min} = 1.9	θ_{max} = 25.7, θ_{min} = 2.5	θ_{max} = 25.7, θ_{min} = 1.9	θ_{max} = 25.7, θ_{min} = 1.8	θ_{max} = 25.7, θ_{min} = 2.5	θ_{max} = 25.7, θ_{min} = 2.5	θ_{max} = 25.7, θ_{min} = 2.2
Range of h, k, l	h = −10→10, k = −10→8, l = −14→12	h = −7→9, k = −10→9, l = −13→13	h = −9→9, k = −10→10, l = −13→10	h = −9→9, k = −10→10, l = −11→13	h = −8→9, k = −10→8, l = −13→13	h = −9→9, k = −8→10, l = −13→13	h = −9→9, k = −10→10, l = −14→14	h = −10→10, k = −18→18, l = −14→14
$R[F^2 > 2\sigma(F^2)]$, $wR(F^2)$, S	0.035, 0.081, 1.05	0.034, 0.078, 1.01	0.030, 0.064, 1.06	0.035, 0.078, 1.07	0.026, 0.052, 1.01	0.054, 0.139, 1.09	0.033, 0.067, 1.01	0.040, 0.110, 1.10
No. of reflections	2908	2585	2717	2631	2693	2802	2854	2952
No. of parameters	154	154	154	154	154	154	154	154
$\Delta\rho_{max}$, $\Delta\rho_{min}$ (e Å^{-3})	0.64, −0.57	0.87, −0.65	0.98, −0.99	0.77, −0.66	0.64, −0.67	1.93, −1.67	1.51, −0.91	3.49, −0.80

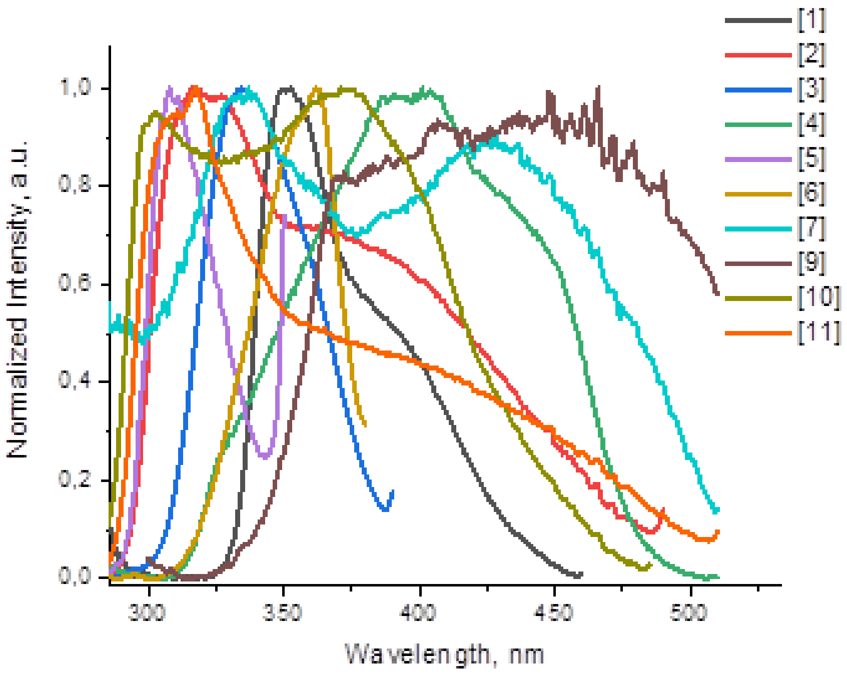

Fig. S13. Normalized excitation spectra of complexes in solid state at 298 K.

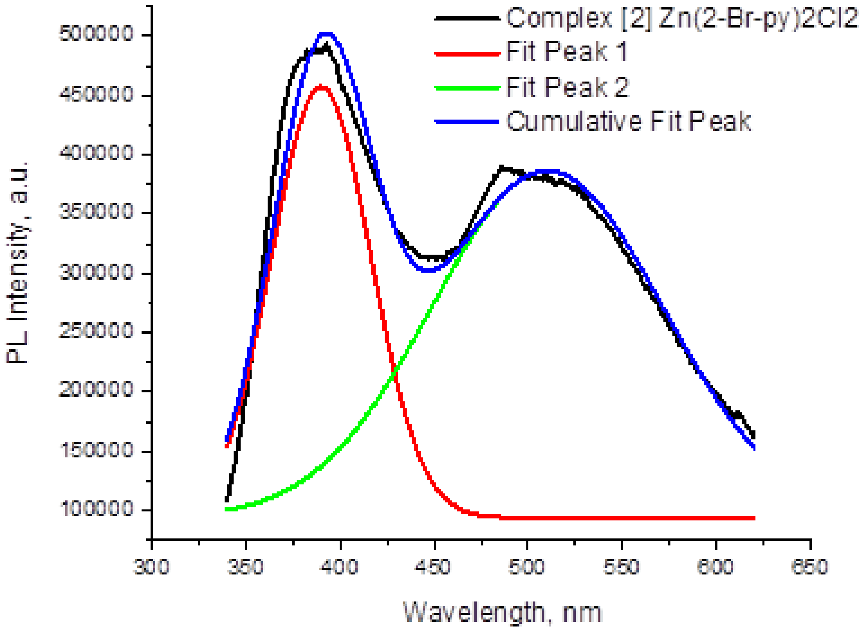

Fig. S14. Emission spectra of **2** and its decomposition on two and three Gauss components, T = 298 K, λex = 350 nm. Gauss fit was performed in eV scale and then spectra were reported in nm scale. Conversion between eV and nm scales was performed with account of λ_2 factor

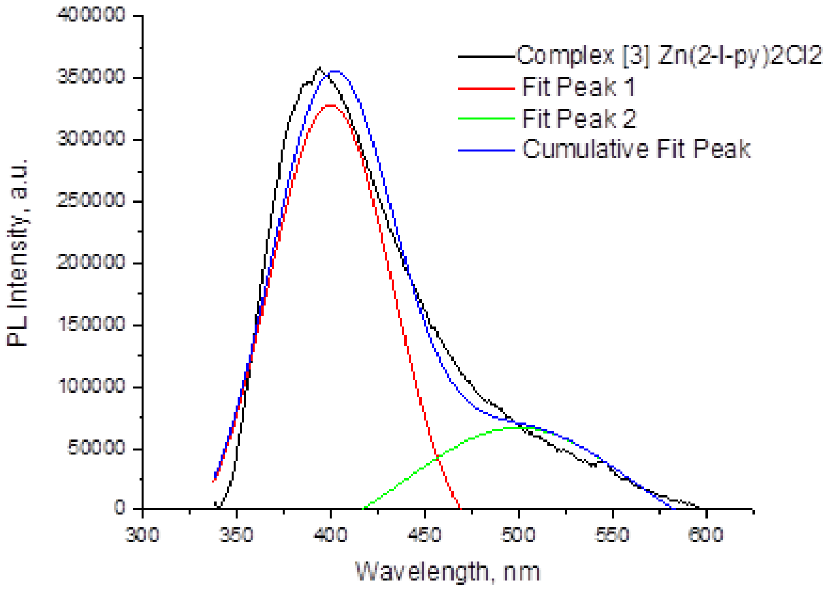

Fig. S15. Description see S14, for **3**

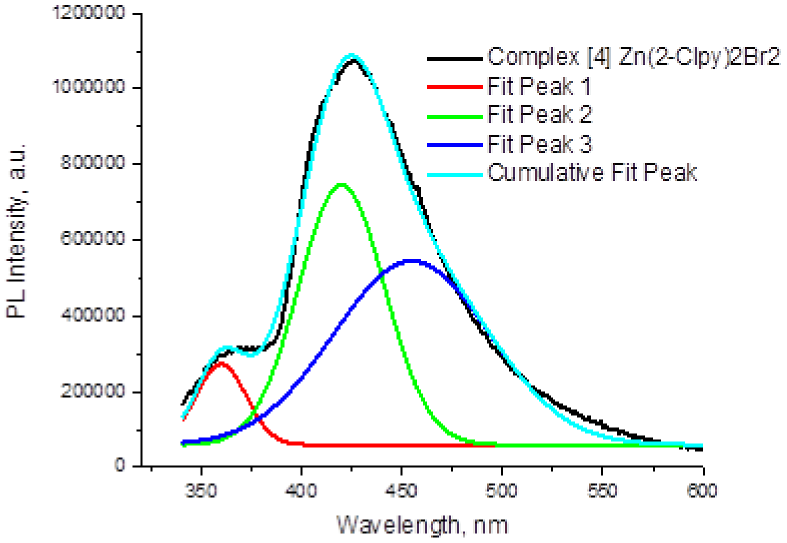

Fig. S16. Description see S14, for **4**

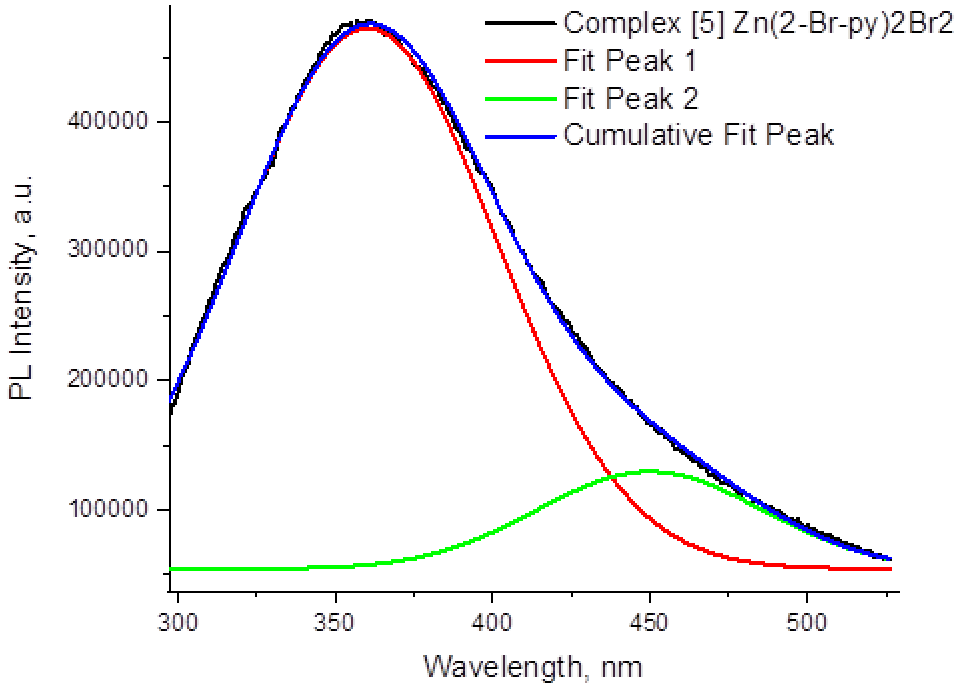

Fig. S17. Description see S14, for **5**

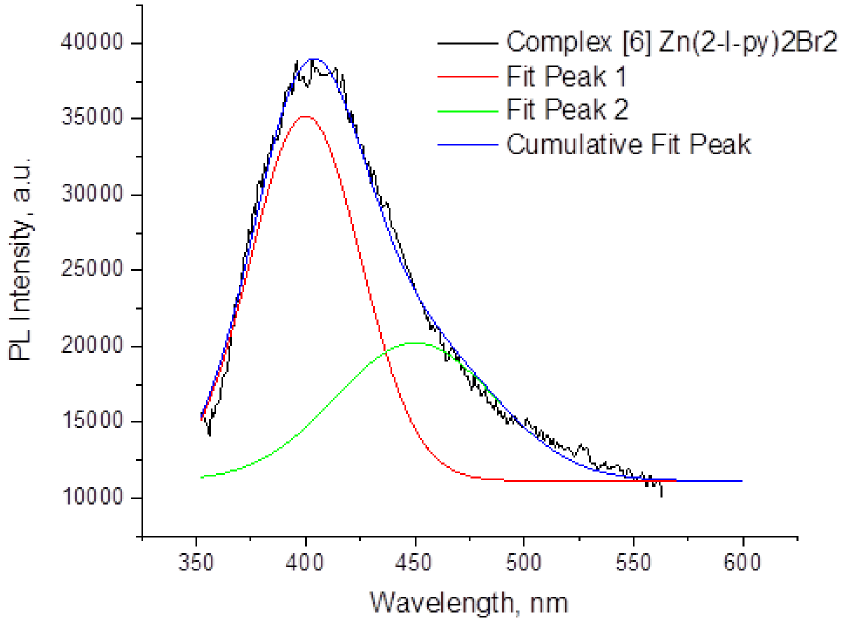

Fig. S18. Description see S14, for **6**

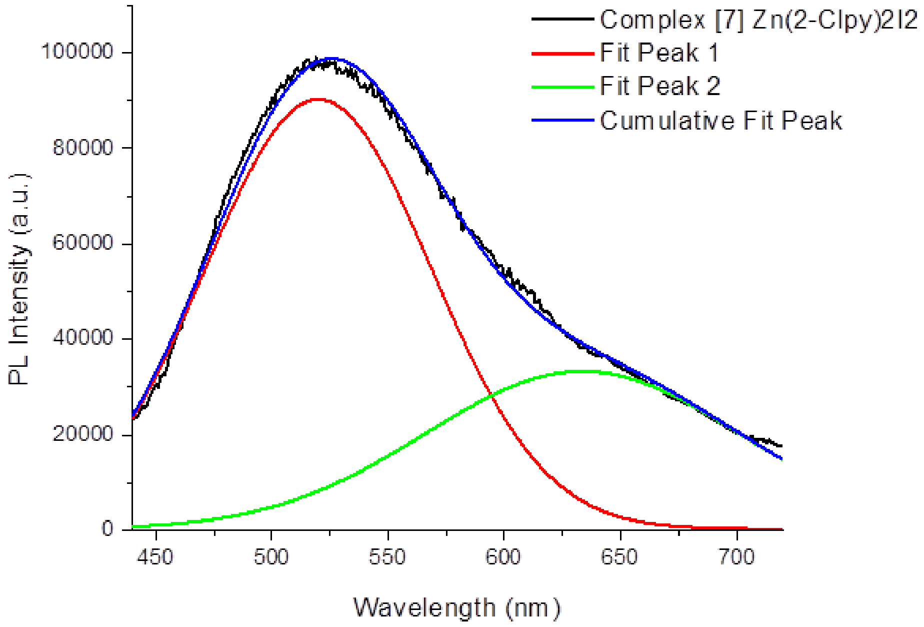

Fig. S19. Description see S14, for **7**

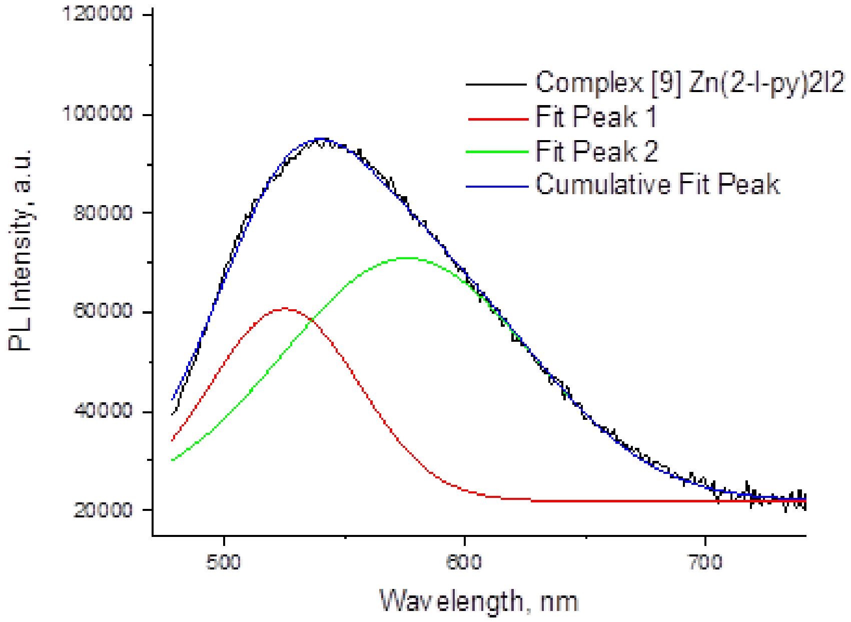

Fig. S20. Description see S14, for **9**

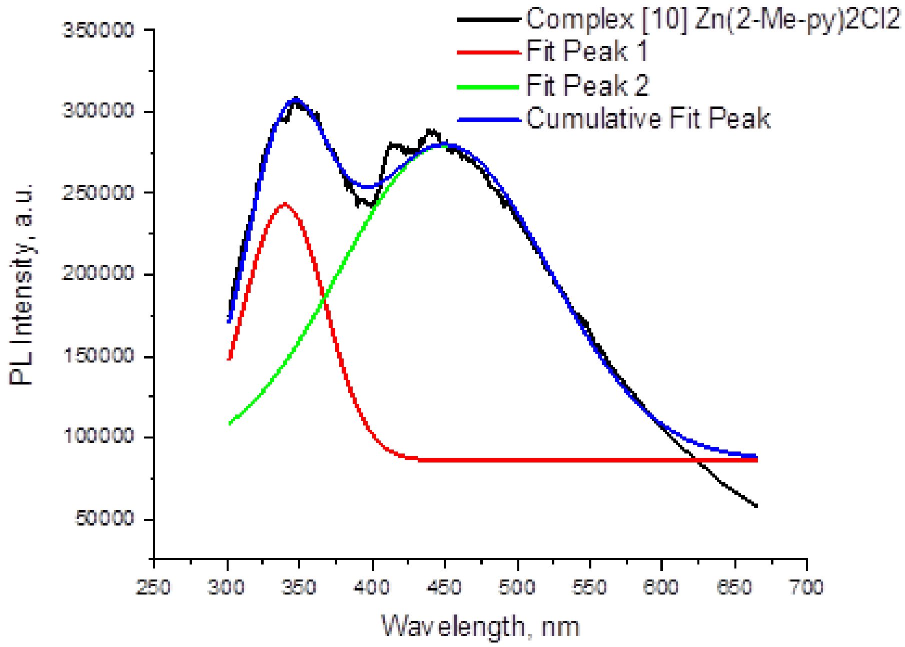

Fig. S21. Description see S14, for **10**

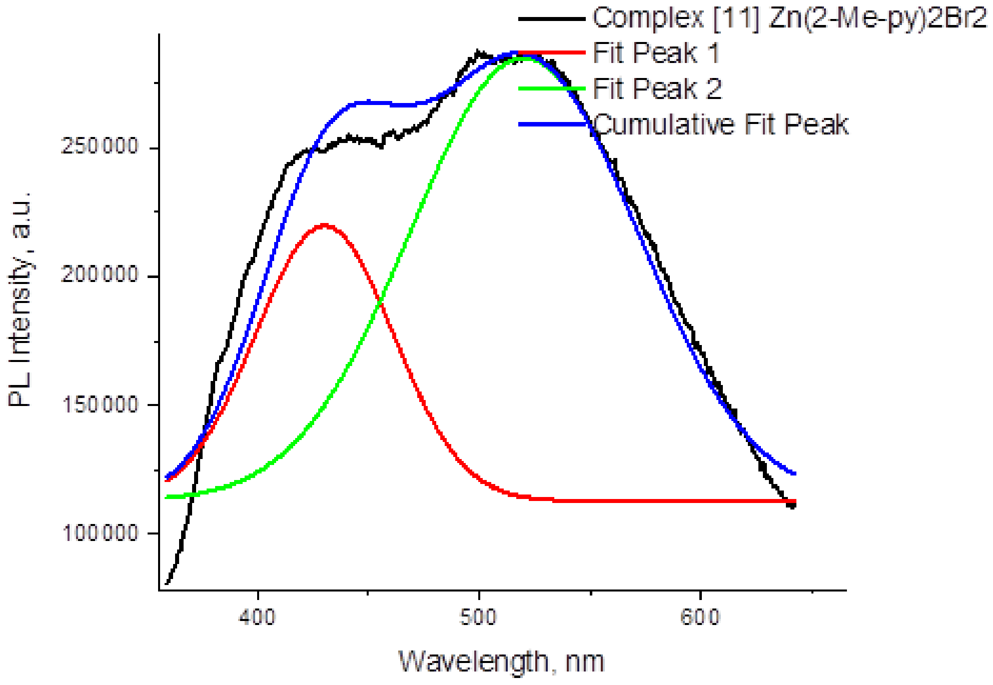

Fig. S22. Description see S14, for **11**

Table 3S. Cartesian atomic coordinates for model supramolecular trimeric associates.

Atom	X	Y	Z
**1**			
C	4.440975	4.226013	3.951839
C	4.121055	5.106585	4.968365
H	3.701437	5.940238	4.788344
C	4.437899	4.720047	6.261930
H	4.258331	5.300021	6.991716
C	5.016528	3.488396	6.481836
H	5.225201	3.205979	7.364693
C	5.287634	2.670387	5.406021
H	5.672115	1.816538	5.566639
C	3.884008	1.773042	0.297520
H	4.619745	2.239740	-0.083004
C	2.763193	1.557765	-0.471073
H	2.728628	1.861821	-1.370100
C	1.689221	0.893307	0.086238
H	0.908952	0.728348	-0.430110
C	1.755268	0.471191	1.397050
H	1.021838	0.032475	1.810990
C	2.917872	0.708678	2.080483
N	5.028145	3.036863	4.138328
N	3.971700	1.340003	1.581382
Cl	6.676803	0.025179	3.583389
Cl	7.048009	2.842922	1.212285
Cl	4.066612	4.653443	2.316882

S-16

Cl	3.057617	0.191137	3.731179
Zn	5.695180	1.787289	2.622271
C	6.526293	3.519445	-3.951839
C	6.846213	2.638873	-4.968365
H	7.265831	1.805220	-4.788344
C	6.529369	3.025411	-6.261930
H	6.708937	2.445437	-6.991716
C	5.950740	4.257062	-6.481836
H	5.742068	4.539478	-7.364693
C	5.679634	5.075071	-5.406021
H	5.295154	5.928919	-5.566639
C	7.083260	5.972416	-0.297520
H	6.347523	5.505717	0.083004
C	8.204075	6.187693	0.471073
H	8.238640	5.883637	1.370100
C	9.278047	6.852151	-0.086238
H	10.058316	7.017110	0.430110
C	9.212000	7.274267	-1.397050
H	9.945430	7.712983	-1.810990
C	8.049396	7.036780	-2.080483
N	5.939123	4.708595	-4.138328
N	6.995568	6.405455	-1.581382
Cl	4.290466	7.720279	-3.583389
Cl	3.919260	4.902536	-1.212285
Cl	6.900656	3.092015	-2.316882
Cl	7.909651	7.554320	-3.731179

Zn	5.272088	5.958169	-2.622271
C	5.856815	-4.194073	6.827864
C	6.176736	-5.074645	5.811338
H	6.596354	-5.908297	5.991359
C	5.859891	-4.688107	4.517774
H	6.039459	-5.268080	3.787988
C	5.281263	-3.456456	4.297868
H	5.072590	-3.174039	3.415010
C	5.010156	-2.638446	5.373682
H	4.625676	-1.784598	5.213065
C	6.413783	-1.741102	10.482184
H	5.678045	-2.207800	10.862707
C	7.534598	-1.525824	11.250777
H	7.569163	-1.829881	12.149804
C	8.608569	-0.861366	10.693466
H	9.388839	-0.696407	11.209814
C	8.542522	-0.439250	9.382654
H	9.275952	-0.000535	8.968713
C	7.379918	-0.676737	8.699221
N	5.269645	-3.004923	6.641375
N	6.326090	-1.308062	9.198321
Cl	3.620988	0.006761	7.196314
Cl	3.249782	-2.810982	9.567418
Cl	6.231178	-4.621502	8.462822
Cl	7.240173	-0.159197	7.048525
Zn	4.602611	-1.755348	8.157433

2			
C	9.227428	6.198175	10.548692
H	8.476952	5.754557	10.925971
C	10.327963	6.409213	11.331357
H	10.331078	6.130059	12.239423
C	11.437225	7.032075	10.786518
H	12.214512	7.183864	11.312980
C	11.394059	7.428911	9.464418
H	12.143282	7.840685	9.050384
C	10.233399	7.208108	8.767154
C	7.900934	5.224971	5.411637
H	7.497124	6.070746	5.257049
C	8.181496	4.428744	4.338174
H	7.960806	4.713601	3.458215
C	8.791566	3.206171	4.543569
H	8.986473	2.634923	3.809550
C	9.112993	2.826513	5.829995
H	9.553631	2.002991	6.004041
C	8.770810	3.688882	6.859136
Cl	6.463964	7.864815	7.222903
Cl	6.096383	5.053692	9.565927
Zn	7.472102	6.117100	8.184803
Br	10.142643	7.756378	6.951348
Br	9.199706	3.207261	8.636133
N	9.163091	6.596760	9.254699
N	8.170308	4.864183	6.680766

C	6.181431	1.837355	11.071910
H	6.931906	2.280973	10.694631
C	5.080895	1.626317	10.289244
H	5.077781	1.905471	9.381179
C	3.971634	1.003455	10.834084
H	3.194347	0.851666	10.307622
C	4.014800	0.606619	12.156183
H	3.265577	0.194845	12.570218
C	5.175460	0.827422	12.853448
C	7.507924	2.810559	16.208965
H	7.911734	1.964784	16.363553
C	7.227363	3.606786	17.282428
H	7.448053	3.321929	18.162387
C	6.617293	4.829359	17.077032
H	6.422386	5.400607	17.811052
C	6.295865	5.209017	15.790607
H	5.855227	6.032539	15.616561
C	6.638049	4.346648	14.761466
Cl	8.944894	0.170715	14.397699
Cl	9.312476	2.981838	12.054675
Zn	7.936757	1.918430	13.435799
Br	5.266215	0.279152	14.669254
Br	6.209153	4.828269	12.984469
N	6.245767	1.438770	12.365903
N	7.238551	3.171347	14.939836
C	6.814104	9.678179	0.261609

H	7.564580	10.121796	-0.115670
C	5.713569	9.467140	-0.521057
H	5.710454	9.746295	-1.429122
C	4.604307	8.844278	0.023783
H	3.827020	8.692489	-0.502679
C	4.647473	8.447442	1.345882
H	3.898250	8.035668	1.759917
C	5.808133	8.668245	2.043147
C	8.140598	10.651383	5.398664
H	8.544408	9.805608	5.553252
C	7.860036	11.447609	6.472127
H	8.080726	11.162752	7.352086
C	7.249966	12.670183	6.266731
H	7.055059	13.241430	7.000751
C	6.928539	13.049840	4.980306
H	6.487901	13.873362	4.806260
C	7.270722	12.187471	3.951165
Cl	9.577568	8.011538	3.587398
Cl	9.945149	10.822661	1.244374
Zn	8.569430	9.759253	2.625498
Br	5.898889	8.119975	3.858953
Br	6.841826	12.669092	2.174168
N	6.878441	9.279593	1.555602
N	7.871224	11.012170	4.129535
**3**			
C	10.104779	7.352647	8.908239

S-21

C	11.251424	7.545026	9.648403
H	12.016716	7.952791	9.259707
C	11.264938	7.136673	10.951353
H	12.042353	7.259331	11.483483
C	10.123648	6.536934	11.496622
H	10.099983	6.261837	12.405402
C	9.042374	6.360199	10.673244
H	8.271346	5.934613	11.029092
C	7.753991	5.378566	5.516189
H	7.314376	6.211723	5.389178
C	8.052587	4.626496	4.419082
H	7.817556	4.931708	3.550814
C	8.698546	3.416211	4.580035
H	8.908065	2.873521	3.828922
C	9.031856	3.016431	5.849043
H	9.489946	2.196329	5.990287
C	8.690733	3.824822	6.919871
N	9.013418	6.757686	9.385623
N	8.055015	4.994568	6.773152
Cl	6.280574	7.980436	7.379846
Cl	5.940787	5.123114	9.639862
Zn	7.340615	6.231935	8.297933
I	10.059554	7.990830	6.910017
I	9.217908	3.236079	8.861486
C	5.156123	0.680859	12.990087
C	4.009478	0.488480	12.249924

H	3.244185	0.080715	12.638619
C	3.995963	0.896833	10.946974
H	3.218549	0.774176	10.414844
C	5.137254	1.496572	10.401705
H	5.160919	1.771669	9.492925
C	6.218527	1.673307	11.225082
H	6.989556	2.098893	10.869235
C	7.506911	2.654940	16.382138
H	7.946525	1.821783	16.509149
C	7.208315	3.407010	17.479244
H	7.443346	3.101798	18.347513
C	6.562355	4.617295	17.318292
H	6.352836	5.159985	18.069404
C	6.229046	5.017075	16.049284
H	5.770956	5.837177	15.908040
C	6.570169	4.208684	14.978456
N	6.247484	1.275820	12.512704
N	7.205887	3.038938	15.125174
Cl	8.980328	0.053070	14.518481
Cl	9.320115	2.910392	12.258464
Zn	7.920287	1.801571	13.600394
I	5.201347	0.042676	14.988310
I	6.042993	4.797427	13.036840
C	5.598535	9.031738	2.040924
C	4.451890	8.839358	1.300761
H	3.686598	8.431594	1.689456

C	4.438376	9.247712	-0.002190
H	3.660961	9.125054	-0.534319
C	5.579666	9.847451	-0.547458
H	5.603331	10.122548	-1.456239
C	6.660940	10.024185	0.275919
H	7.431968	10.449772	-0.079929
C	7.949323	11.005818	5.432975
H	8.388938	10.172662	5.559985
C	7.650728	11.757889	6.530081
H	7.885758	11.452676	7.398350
C	7.004768	12.968174	6.369128
H	6.795249	13.510864	7.120241
C	6.671458	13.367954	5.100120
H	6.213368	14.188056	4.958876
C	7.012581	12.559563	4.029292
N	6.689896	9.626699	1.563541
N	7.648299	11.389817	4.176011
Cl	9.422740	8.403948	3.569318
Cl	9.762527	11.261270	1.309301
Zn	8.362699	10.152450	2.651230
I	5.643760	8.393555	4.039146
I	6.485406	13.148306	2.087677
**4**			
C	3.857053	1.811724	0.376262
H	4.557030	2.345521	0.017552
C	2.788490	1.483434	-0.436596

S-24

H	2.758057	1.774498	-1.340503
C	1.768433	0.724140	0.092146
H	1.024207	0.482990	-0.446469
C	1.832970	0.317855	1.404127
H	1.136759	-0.200861	1.790262
C	2.936379	0.683668	2.140197
C	4.445069	4.293449	4.047835
C	4.139586	5.154671	5.080088
H	3.734014	5.996207	4.906766
C	4.437029	4.767527	6.365742
H	4.250336	5.343465	7.098521
C	5.011163	3.527183	6.576361
H	5.211107	3.230584	7.456135
C	5.282593	2.739244	5.504617
H	5.676818	1.888188	5.657097
N	3.944225	1.406287	1.653140
N	5.019228	3.106463	4.237612
Zn	5.665375	1.873596	2.696802
Br	7.135393	2.947455	1.196470
Br	6.714777	-0.019656	3.667734
Cl	3.044867	0.221544	3.801236
Cl	4.060476	4.726146	2.425410
C	7.023861	6.147377	-0.376262
H	6.323884	5.613580	-0.017552
C	8.092424	6.475667	0.436596
H	8.122857	6.184603	1.340503

C	9.112481	7.234961	-0.092146
H	9.856707	7.476112	0.446469
C	9.047944	7.641247	-1.404127
H	9.744155	8.159962	-1.790262
C	7.944535	7.275434	-2.140197
C	6.435845	3.665652	-4.047835
C	6.741328	2.804430	-5.080088
H	7.146900	1.962894	-4.906766
C	6.443885	3.191575	-6.365742
H	6.630578	2.615637	-7.098521
C	5.869752	4.431918	-6.576361
H	5.669807	4.728517	-7.456135
C	5.598321	5.219857	-5.504617
H	5.204096	6.070913	-5.657097
N	6.936689	6.552814	-1.653140
N	5.861686	4.852638	-4.237612
Zn	5.215539	6.085505	-2.696802
Br	3.745521	5.011647	-1.196470
Br	4.166137	7.978757	-3.667734
Cl	7.836047	7.737557	-3.801236
Cl	6.820438	3.232955	-2.425410
C	6.518825	-1.828140	10.593481
H	5.818848	-2.361937	10.952192
C	7.587388	-1.499850	11.406339
H	7.617821	-1.790914	12.310246
C	8.607445	-0.740556	10.877598

H	9.351670	-0.499405	11.416212
C	8.542908	-0.334270	9.565616
H	9.239119	0.184445	9.179481
C	7.439499	-0.700084	8.829547
C	5.930809	-4.309865	6.921908
C	6.236291	-5.171087	5.889655
H	6.641864	-6.012623	6.062977
C	5.938849	-4.783942	4.604001
H	6.125541	-5.359881	3.871222
C	5.364715	-3.543599	4.393382
H	5.164771	-3.247000	3.513609
C	5.093285	-2.755660	5.465126
H	4.699060	-1.904604	5.312647
N	6.431653	-1.422703	9.316603
N	5.356650	-3.122879	6.732132
Zn	4.710503	-1.890012	8.272942
Br	3.240485	-2.963870	9.773274
Br	3.661101	0.003240	7.302010
Cl	7.331011	-0.237960	7.168508
Cl	6.315402	-4.742562	8.544333
**5**			
C	5.249298	2.787050	5.493683
H	5.669805	1.948454	5.641743
C	4.945073	3.576799	6.574299
H	5.144021	3.280903	7.454923
C	4.349554	4.804119	6.370993

H	4.154689	5.369443	7.109082
C	4.039018	5.202171	5.092595
H	3.615767	6.035828	4.924647
C	4.363642	4.343250	4.059491
C	2.902684	0.751208	2.107090
C	1.799474	0.409385	1.359057
H	1.078967	-0.076987	1.743570
C	1.771696	0.791925	0.044197
H	1.022693	0.578618	-0.499426
C	2.848458	1.494670	-0.487272
H	2.854210	1.754894	-1.401044
C	3.897768	1.801335	0.332582
H	4.622117	2.302371	-0.024308
N	4.971298	3.161742	4.224125
N	3.951315	1.422750	1.630868
Zn	5.640298	1.923384	2.704965
Br	3.926864	4.827488	2.286641
Br	2.971437	0.255045	3.933309
Br	7.146221	3.014103	1.242045
Br	6.716264	0.038637	3.678514
C	5.042331	-2.732811	5.555559
H	4.621824	-1.894215	5.407499
C	5.346556	-3.522560	4.474943
H	5.147608	-3.226664	3.594318
C	5.942075	-4.749880	4.678249
H	6.136940	-5.315204	3.940160

C	6.252611	-5.147932	5.956646
H	6.675862	-5.981589	6.124595
C	5.927987	-4.289011	6.989750
C	7.388945	-0.696969	8.942151
C	8.492155	-0.355146	9.690185
H	9.212662	0.131227	9.305671
C	8.519933	-0.737686	11.005045
H	9.268936	-0.524379	11.548667
C	7.443171	-1.440431	11.536513
H	7.437419	-1.700655	12.450285
C	6.393861	-1.747096	10.716659
H	5.669512	-2.248131	11.073550
N	5.320331	-3.107503	6.825117
N	6.340314	-1.368511	9.418374
Zn	4.651331	-1.869145	8.344277
Br	6.364765	-4.773249	8.762601
Br	7.320192	-0.200806	7.115933
Br	3.145408	-2.959864	9.807196
Br	3.575365	0.015602	7.370728
C	5.571691	5.283366	-5.493683
H	5.151184	6.121963	-5.641743
C	5.875915	4.493617	-6.574299
H	5.676968	4.789514	-7.454923
C	6.471435	3.266298	-6.370993
H	6.666300	2.700974	-7.109082
C	6.781970	2.868246	-5.092595

H	7.205222	2.034589	-4.924647
C	6.457346	3.727167	-4.059491
C	7.918305	7.319209	-2.107090
C	9.021515	7.661031	-1.359057
H	9.742021	8.147404	-1.743570
C	9.049293	7.278492	-0.044197
H	9.798295	7.491798	0.499426
C	7.972531	6.575746	0.487272
H	7.966779	6.315522	1.401044
C	6.923221	6.269081	-0.332582
H	6.198872	5.768046	0.024308
N	5.849691	4.908675	-4.224125
N	6.869674	6.647667	-1.630868
Zn	5.180691	6.147032	-2.704965
Br	6.894125	3.242929	-2.286641
Br	7.849552	7.815372	-3.933309
Br	3.674767	5.056314	-1.242045
Br	4.104724	8.031780	-3.678514
**6**			
C	7.797884	5.492932	5.690246
H	7.372512	6.332044	5.558751
C	8.093858	4.715208	4.593331
H	7.858172	5.008787	3.721194
C	8.734183	3.512797	4.769782
H	8.922375	2.956498	4.023520
C	9.101232	3.118733	6.042023

S-30

H	9.589030	2.316054	6.184757
C	8.736648	3.932004	7.102974
C	10.169601	7.492761	9.154070
C	11.249402	7.752773	9.920561
H	12.011688	8.177739	9.544059
C	11.243026	7.398520	11.259112
H	11.982937	7.593720	11.821056
C	10.140318	6.761800	11.743508
H	10.108684	6.500550	12.656105
C	9.081613	6.496760	10.927565
H	8.325713	6.043627	11.282714
N	9.079286	6.860994	9.628350
N	8.098799	5.091432	6.972603
Zn	7.413679	6.325663	8.502552
Br	6.299316	8.221569	7.569837
Br	5.869596	5.183277	9.899657
I	10.153523	8.056804	7.123091
I	9.288502	3.349235	9.051571
C	7.560933	2.811686	16.787519
H	7.986304	1.972574	16.919013
C	7.264959	3.589410	17.884433
H	7.500644	3.295831	18.756571
C	6.624634	4.791821	17.707983
H	6.436441	5.348120	18.454245
C	6.257584	5.185884	16.435741
H	5.769786	5.988564	16.293008

S-31

C	6.622168	4.372614	15.374791
C	5.189215	0.811857	13.323695
C	4.109415	0.551845	12.557203
H	3.347129	0.126879	12.933706
C	4.115790	0.906097	11.218652
H	3.375879	0.710897	10.656708
C	5.218498	1.542818	10.734256
H	5.250132	1.804068	9.821659
C	6.277204	1.807857	11.550199
H	7.033104	2.260991	11.195051
N	6.279530	1.443624	12.849414
N	7.260018	3.213186	15.505162
Zn	7.945137	1.978955	13.975213
Br	9.059500	0.083049	14.907928
Br	9.489220	3.121341	12.578108
I	5.205293	0.247813	15.354673
I	6.070314	4.955382	13.426194
C	7.985016	11.074675	5.548636
H	8.410388	10.235563	5.680131
C	7.689042	11.852399	6.645551
H	7.924728	11.558819	7.517688
C	7.048717	13.054810	6.469101
H	6.860524	13.611109	7.215362
C	6.681668	13.448873	5.196859
H	6.193869	14.251553	5.054125
C	7.046252	12.635603	4.135909

C	5.613299	9.074846	2.084813
C	4.533498	8.814834	1.318321
H	3.771212	8.389868	1.694823
C	4.539874	9.169086	-0.020230
H	3.799962	8.973886	-0.582174
C	5.642581	9.805807	-0.504626
H	5.674215	10.067057	-1.417223
C	6.701287	10.070846	0.311317
H	7.457187	10.523980	-0.043832
N	6.703614	9.706613	1.610532
N	7.684101	11.476175	4.266280
Zn	8.369220	10.241943	2.736331
Br	9.483583	8.346038	3.669046
Br	9.913303	11.384330	1.339225
I	5.629376	8.510802	4.115791
I	6.494398	13.218371	2.187311
**7**			
C	8.705490	3.755197	7.245338
C	9.037169	2.889639	6.225801
H	9.442569	2.049775	6.405249
C	8.760255	3.287223	4.935379
H	8.954320	2.711963	4.205016
C	8.202177	4.520621	4.714784
H	8.038470	4.823957	3.828976
C	7.882810	5.312279	5.793756
H	7.473633	6.154379	5.633739

C	10.108468	7.511188	9.213546
C	11.123923	8.019302	9.995343
H	11.769769	8.614792	9.635305
C	11.168798	7.631204	11.322340
H	11.852507	7.961804	11.893829
C	10.215348	6.762320	11.811535
H	10.240752	6.474529	12.715631
C	9.230156	6.321536	10.964588
H	8.569662	5.727163	11.302910
N	8.125882	4.943895	7.075034
N	9.158718	6.699346	9.661593
Cl	9.099169	3.330256	8.876825
Cl	10.039235	7.922156	7.532911
Zn	7.451576	6.164871	8.605024
I	5.803666	4.999849	10.189763
I	6.286989	8.225650	7.577944
C	6.936491	12.710628	4.184443
C	6.604812	13.576185	5.203979
H	6.199412	14.416050	5.024531
C	6.881726	13.178601	6.494401
H	6.687661	13.753862	7.224764
C	7.439804	11.945203	6.714996
H	7.603511	11.641867	7.600804
C	7.759171	11.153545	5.636025
H	8.168348	10.311445	5.796042
C	5.533513	8.954636	2.216234

C	4.518058	8.446523	1.434437
H	3.872212	7.851032	1.794476
C	4.473183	8.834620	0.107440
H	3.789473	8.504020	-0.464049
C	5.426633	9.703504	-0.381755
H	5.401228	9.991295	-1.285850
C	6.411825	10.144289	0.465192
H	7.072319	10.738661	0.126871
N	7.516099	11.521929	4.354746
N	6.483263	9.766478	1.768187
Cl	6.542811	13.135569	2.552956
Cl	5.602746	8.543668	3.896869
Zn	8.190405	10.300953	2.824756
I	9.838315	11.465975	1.240017
I	9.354992	8.240174	3.851836
C	6.722373	4.251091	15.614223
C	6.390694	5.116649	16.633759
H	5.985294	5.956513	16.454312
C	6.667608	4.719064	17.924182
H	6.473543	5.294325	18.654545
C	7.225686	3.485667	18.144776
H	7.389393	3.182331	19.030584
C	7.545053	2.694009	17.065805
H	7.954230	1.851909	17.225822
C	5.319395	0.495099	13.646015
C	4.303940	-0.013014	12.864218

H	3.658094	-0.608504	13.224256
C	4.259065	0.375084	11.537220
H	3.575356	0.044484	10.965731
C	5.212515	1.243968	11.048026
H	5.187111	1.531759	10.143930
C	6.197707	1.684752	11.894972
H	6.858201	2.279124	11.556651
N	7.301981	3.062393	15.784527
N	6.269145	1.306942	13.197967
Cl	6.328694	4.676032	13.982736
Cl	5.388628	0.084132	15.326650
Zn	7.976287	1.841417	14.254536
I	9.624197	3.006438	12.669797
I	9.140874	-0.219363	15.281616
**9**			
C	3.496774	9.503204	4.837996
C	2.767366	8.753988	5.727893
H	2.108012	8.135203	5.437993
C	3.044812	8.952667	7.076401
H	2.580379	8.461279	7.744121
C	4.667520	10.610343	6.425315
H	5.328846	11.244295	6.677195
C	6.026201	10.692242	1.108509
H	5.306040	11.268561	0.880392
C	6.686753	10.021891	0.093861
H	6.435124	10.156871	-0.812669

C	7.710687	9.158929	0.411087
H	8.163268	8.672090	-0.267325
C	8.069638	9.014849	1.742962
H	8.786918	8.444596	1.992466
C	7.367797	9.714015	2.699393
C	4.008594	9.877811	7.425707
H	4.225291	10.017341	8.339366
N	4.384102	10.435931	5.106510
N	6.365240	10.558778	2.414246
Zn	5.340444	11.722943	3.786398
I	3.075847	9.175915	2.749769
I	7.844200	9.430102	4.734631
I	6.930768	13.349984	5.013837
I	3.627290	13.198018	2.519750
C	-0.626067	13.246246	-1.102569
C	-1.355475	13.995462	-0.212672
H	-2.014830	14.614247	-0.502572
C	-1.078029	13.796783	1.135836
H	-1.542463	14.288171	1.803556
C	0.544678	12.139107	0.484750
H	1.206005	11.505155	0.736630
C	1.903360	12.057209	-4.832056
H	1.183199	11.480889	-5.060174
C	2.563912	12.727559	-5.846704
H	2.312283	12.592579	-6.753235
C	3.587846	13.590521	-5.529478

H	4.040427	14.077360	-6.207891
C	3.946797	13.734601	-4.197603
H	4.664077	14.304854	-3.948100
C	3.244956	13.035435	-3.241172
C	-0.114247	12.871639	1.485141
H	0.102450	12.732109	2.398800
N	0.261261	12.313519	-0.834055
N	2.242399	12.190672	-3.526320
Zn	1.217603	11.026507	-2.154168
I	-1.046994	13.573535	-3.190796
I	3.721359	13.319348	-1.205935
I	2.807927	9.399466	-0.926728
I	-0.495551	9.551432	-3.420815
C	7.619615	13.246246	10.778562
C	6.890207	13.995462	11.668458
H	6.230853	14.614247	11.378559
C	7.167653	13.796783	13.016967
H	6.703220	14.288171	13.684686
C	8.790361	12.139107	12.365881
H	9.451687	11.505155	12.617761
C	10.149042	12.057209	7.049075
H	9.428881	11.480889	6.820957
C	10.809594	12.727559	6.034426
H	10.557965	12.592579	5.127896
C	11.833528	13.590521	6.351652
H	12.286109	14.077360	5.673240

C	12.192479	13.734601	7.683527
H	12.909759	14.304854	7.933031
C	11.490638	13.035435	8.639958
C	8.131435	12.871639	13.366272
H	8.348132	12.732109	14.279931
N	8.506943	12.313519	11.047075
N	10.488081	12.190672	8.354811
Zn	9.463285	11.026507	9.726963
I	7.198688	13.573535	8.690334
I	11.967041	13.319348	10.675196
I	11.053609	9.399466	10.954402
I	7.750131	9.551432	8.460315

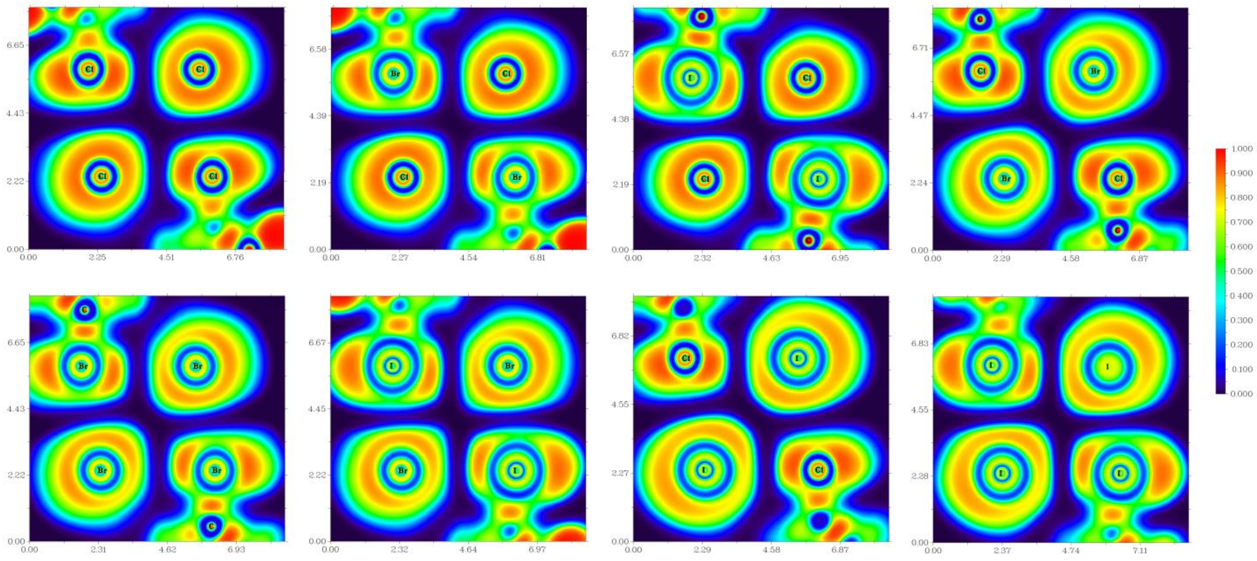

Figure S23. Visualization of electron localization function (ELF) in the areas of intermolecular non-covalent interactions X···Y (X, Y = Cl, Br, I) in **1–7** and **9** (from left to right, from top to bottom). Length units – Å, the color scale for the ELF maps presented in a.u.
